# Marine Bioactives as Functional Food Ingredients: Potential to Reduce the Incidence of Chronic Diseases

**DOI:** 10.3390/md9061056

**Published:** 2011-06-14

**Authors:** Sinéad Lordan, R. Paul Ross, Catherine Stanton

**Affiliations:** Teagasc Food Research Centre Moorepark, Fermoy, Co. Cork, Ireland; E-Mails: sinead.lordan@teagasc.ie (S.L.); paul.ross@teagasc.ie (R.P.R.)

**Keywords:** disease, functional food ingredients, marine, polyunsaturated fatty acids

## Abstract

The marine environment represents a relatively untapped source of functional ingredients that can be applied to various aspects of food processing, storage, and fortification. Moreover, numerous marine-based compounds have been identified as having diverse biological activities, with some reported to interfere with the pathogenesis of diseases. Bioactive peptides isolated from fish protein hydrolysates as well as algal fucans, galactans and alginates have been shown to possess anticoagulant, anticancer and hypocholesterolemic activities. Additionally, fish oils and marine bacteria are excellent sources of omega-3 fatty acids, while crustaceans and seaweeds contain powerful antioxidants such as carotenoids and phenolic compounds. On the basis of their bioactive properties, this review focuses on the potential use of marine-derived compounds as functional food ingredients for health maintenance and the prevention of chronic diseases.

## 1. Introduction

Increasing knowledge regarding the impact of diet on human health along with state-of-the-art technologies has led to significant nutritional discoveries, product innovations, and mass production on an unprecedented scale [1]. In particular, naturally occurring bioactive extracts or single compounds thereof, that are believed to benefit human health, have spawned an important and dynamic new area of research resulting in substantial advances in nutritional knowledge. There is also growing awareness that dietary source and form of food may affect overall health. Suitably, the role of food as an agent for improving health has been recognised, initiating the development of new classes of food, known as functional foods [2].

The concept of functional foods is to improve the general conditions of the body and decrease the risk of illness and disease [3]. That is to say, bioactive compounds present as natural constituents or as fortificants in food having the potential to provide health benefits beyond the basic nutritional value of the product. Marine-derived nutrients and other marine bioactive components have excellent potential as functional food ingredients as they possess advantageous physiological effects, with medicinal characteristics and added health benefits such as anticancer or anti-inflammatory activity.

The marine world, due to its phenomenal biodiversity, is a rich natural resource of many biologically active compounds such as polyunsaturated fatty acids (PUFAs), sterols, proteins, polysaccharides, antioxidants and pigments. Many marine organisms live in complex habitats exposed to extreme conditions and, in adapting to new environmental surroundings, they produce a wide variety of secondary (biologically active) metabolites which cannot be found in other organisms. Moreover, considering its great taxonomic diversity, investigations related to the search of new bioactive compounds from the marine environment can be seen as an almost unlimited field [4,5].

Marine-based bioactive food ingredients can be derived from a vast array of sources, including marine plants, microorganisms, and sponges, all of which contain their own unique set of biomolecules [4]. However, proving that these naturally occurring bioactive substances have a defined health benefit poses a dilemma in nutritional research as investigating preventive activity can be difficult when effect is only moderate. This means that the effect of the compounds on the human body may be very small over relatively short periods but could contribute significantly to health when they are consumed throughout life as part of the daily diet [1]. Therefore, to facilitate discussion of this issue, the following review examines the existing scientific knowledge which demonstrates the suitability of marine-derived bioactive compounds as functional food ingredients for the prevention and treatment of chronic diseases.

## 2. Sources of Marine Functional Food Ingredients

### 2.1. Macroalgae

Marine algae are simple chlorophyll containing organisms composed of one cell or grouped together in colonies or as organisms with many cells, sometimes collaborating together as simple tissues. These unicellular or multicellular vegetative organisms do not have true roots or stems and vary greatly in size and morphology—from organisms 3–10 μm in length to giant kelps up to 70 m long and growing up to 50 cm per day. Correspondingly, algae can be classified into two major groups according to their size: macroalgae or microalgae [6,7].

Macroalgae are more commonly known as seaweeds and several characteristics are used to classify them including the nature of their chlorophyll, their cell wall chemistry, and the presence or absence of flagella. However, the feature most commonly employed in algal classification is the presence of specific pigments, other than chlorophyll, which clearly identify macroalgae as belonging to one of three algal divisions. In accordance with this criterion, macroalgae can be classified as brown algae (Phaeophyceae), red algae (Rhodophyceae), or green algae (Chlorophyceae). The presence of these different phytopigments in algae is related to their sea habitat because not all macroalgae need the same light intensity to perform photosynthesis. Thus, green macroalgae, which are able to absorb large amounts of light energy, abound in coastal waters, while red and brown macroalgae prevail at greater depths where penetration of sunlight is limited [6].

Macroalgae are a source of biologically active phytochemicals, which include carotenoids, phycobilins, fatty acids, polysaccharides, vitamins, sterols, tocopherol and phycocyanins among others. Many of these compounds are known to possess biological activity and hence have potential beneficial use in healthcare [8]. However, the chemical and nutritional composition of seaweeds depends on many factors, including species, geographical origin or area of cultivation, seasonal, environmental, and physiological variations, time of harvest, water temperature, and processing methods [6,9–11]. For example, a seasonal variation of protein content of *Palmaria palmata* was observed, with maximum values (approximately 21%) occurring during the winter-spring period and lower levels (12%) during the summer-early autumn period [12].

#### 2.1.1. Proteins, Peptides and Amino Acids

The protein content of macroalgae varies greatly from phylum to phylum [9]. Generally, the protein fraction of brown seaweeds is low (3–15% of dry weight) compared with that of the green or red seaweeds (10–47% of dry weight) [10]. The protein in macroalgae contains all essential amino acids, however, variations in their concentrations are known to occur [12]. Leucine, valine, and methionine are abundant essential amino acids of *Palmaria palmata* and their average levels are close to those generally reported for ovalbumin. On the other hand, isoleucine and threonine concentrations are similar to those recorded for legume proteins [6]. Leucine, phenylalanine and valine are the major essential amino acids of *Ulva rigida*, while levels of histidine, which is an essential amino acid in children, are similar to those found in legumes and eggs [13].

Recently, much attention has been paid to unraveling the structural, compositional and sequential properties of bioactive peptides. Marine bioactive peptides may be produced by one of three methods; solvent extraction, enzymatic hydrolysis or microbial fermentation of marine proteins. However, particularly in food and pharmaceutical industries, the enzymatic hydrolysis method is preferred on account of lack of residual organic solvents or toxic chemicals in the products. Bioactive peptides usually contain 3–20 amino acid residues and their activities are based on their amino acid composition and sequence [14,15]. These peptides are reported to be involved in various biological functions such as antihypertension, immunomodulatory, antithrombotic, antioxidant, anticancer and antimicrobial activities, in addition to nutrient utilisation [15,16].

Among the algal proteins, it is worth noting the occurrence of protein-pigment complexes called phycobiliproteins, some of which are currently used as fluorescent markers in the fields of clinical diagnosis and biotechnological applications [17,18]. Recent studies have shown that phycobiliproteins, which generally make up 1–10% of dry weight of algal biomass, impart antioxidant properties which could be beneficial in the prevention or treatment of several diseases [17]. Moreover, in some countries, phycobiliproteins are utilised as natural food colourings in products such as chewing gums, dairy products, jellies and ice sherbets [4].

Of note, the *in vivo* digestibility of algal proteins is poorly described, and available studies about their assimilation by humans have not provided conclusive results. Nonetheless, several studies have described a high rate of algal protein degradation *in vitro* by proteolytic enzymes such as pepsin, pancreatin and pronase. For instance, the *in vitro* digestibility of proteins from the red seaweed *Porphyra tenera* is approximately 70%. There is a possibility, however, that the high phenolic content of some algae may limit protein availability *in vivo* [9].

#### 2.1.2. Fatty Acids

The lipid content of macroalgae represents only 1–5%, thus its contribution as a food energy source appears to be low [17]. However, PUFAs account for almost half of this lipid fraction, with much of it occurring in the form of omega-3 (n-3) and omega-6 (n-6) fatty acids such as eicosapentanoic acid (EPA) and arachidonic acid (AA) [19]. PUFAs regulate a wide range of functions in the body including blood pressure, blood clotting, and correct development and functioning of the brain and nervous systems [20]. Furthermore, PUFAs have a role in regulating inflammatory responses through the production of inflammatory mediators termed eicosanoids [21].

The n-3 to n-6 ratio of macroalgae is closely matched which may add to their efficacy as a dietary supplement or as part of a balanced diet [22]. Moreover, they contain many essential fatty acids. Red and brown algae, for instance, are particularly rich in the n-3 fatty acids, EPA and α-linolenic acid, and the n-6 fatty acids, AA and linoleic acid, along with relatively high levels of oleic and palmitic acids [11]. In contrast, green seaweeds, like *Ulva pertusa*, are characterised by the presence of hexadecatetraenoic (n-3), oleic and palmitic acids [23]. The n-3 fatty acid, octadecatetraenoic acid, is abundant in *Laminaria* sp. and *Undaria pinnatifida* while hexadecatetraenoic acid is prominent in *Ulva* sp. [6,24].

In addition to fatty acids, the unsaponifiable fraction of macroalgae contains carotenoids (such as β-carotene, lutein and violaxanthin in red and green seaweeds, fucoxanthin in brown seaweeds), tocopherols, sterols (such as fucosterol in brown seaweeds) and terpenoids [25–28].

#### 2.1.3. Polysaccharides

Although algal carbohydrate content is relatively high, macroalgae cannot be considered a potential energy rich food as digestibility of these carbohydrates is low [6]. Moreover, the carbohydrate type varies greatly between algae species. Typical polysaccharides in red algae varieties consist of floridean starch, cellulose, xylan and mannan, and the water soluble fibre fraction is formed by sulfur containing galactans such as agar and carrageenan. Standard polysaccharides in brown algae are fucoidan, laminaran, cellulose, alginates and mannitol whilst the fibres are mainly cellulose and insoluble alginates. Most of these polysaccharides are not digestible by the human gastrointestinal tract and, therefore, can be regarded as dietary fibres [11]. The total dietary fibre content of seaweeds ranges from 29.3–62.3 g/100 g [11,19,29], and so is higher than the fibre content of most fruits and vegetables. Human consumption of algal fibre has been proven to be health promoting and its benefits are well documented [13,30,31].

Storage polysaccharides, such as agar, carrageenans and alginates, are the most commercially exploited components in seaweeds. These storage polysaccharides exhibit textural and stabilizing properties [19]; thus they are used in food applications such as thickening aqueous solutions, forming gels, forming water soluble films and stabilizing products such as ice-cream [4].

Fucoidans are a complex series of sulfated polysaccharides found widely in the cell walls of brown macroalgae. Fucoidans are reported to display numerous physiological and biological properties, including anticoagulant, antiviral, antithrombotic, antitumor and antioxidant activities, as well as having an effect on the inflammatory and immune systems [32,33]. In addition, the therapeutic potential of fucoidans increases with their degree of sulfation and they can be easily extracted using either hot water or an acid solution [32]. Another sulfated polysaccharide, porphyran, makes up the main components of the red macroalga, *Porphyra* [30]. This polysaccharide has reported uses as a gelling agent, a nutritional supplement and as an antioxidant [34]. Alternatively, laminarin, the second major storage glucan in brown algae, has been identified as a modulator of intestinal metabolism through its effects on mucus composition, intestinal pH and short chain fatty acid production [34–36].

Another group of carbohydrate derivatives, oligosaccharides, are commonly defined as carbohydrate molecules with a low degree of polymerisation. Oligosaccharides can be produced naturally or may be derived from algal polysaccharides after chemical, physical or biochemical degradations. To date, numerous oligosaccharides with immunostimulation activities as well as antioxidant and antitumor properties have been characterised. Moreover, oligosaccharides can be beneficial to health when they are added to the diet to enhance the growth of prebiotic bacteria. In this case, oligomers that resist the digestive process are used as a specific substrate for the growth of health beneficial bacteria [37]. For instance, xylo-oligosaccharides and fructo-oligosaccharides are non-digestible oligomers that cannot be absorbed in the gastrointestinal tract. Hence, they are intact in the large bowel and are used as a preferential substrate by anaerobic bacteria such as bifidobacteria and lactobacilli [38,39]. Interestingly, no specific conformation is correlated to the non-digestible oligosaccharide’s biological activity, whereas the immunostimulating, antioxidant, antiangiogenic and antithrombotic activities of poly/oligosaccharides molecules are determined by glycan conformation [37].

While algal polysaccharides have yet to be exploited in the food industry, the fact that they are easy to isolate and have numerous health benefits gives them the potential to serve as valuable bioactive ingredients in functional foods [4].

#### 2.1.4. Vitamins, Minerals and Antioxidants

One of the principal nutritive characteristics of seaweeds is their high antioxidant content (Table 1). In addition, vitamin B_12_ is found in red macroalgae (e.g., *Palmaria longat* and *Porphyra tenera*) and in certain green seaweeds [9]. Red and brown algae contain high levels of folic acid and folate derivatives including 5-metil-tetrahydro-folate, 5-formyl-tetrahydro-folate and tetrahydro-folate. Indeed, amounts as high as 150 μg folic acid per 100 g of dry *Undaria pinnatifida* algae have been detected [40]. As well as seasonal, environmental and physiological variations, vitamin content also depends on the type of seaweed processing. For example, the content of α-tocopherol in *Himanthalia longate* dehydrated (33.3 μg/g dry weight) was considerably higher than in canned *Himanthalia longate* (12 μg/g dry weight) [41].

Seaweeds also contain an incomparable wealth of minerals and trace elements which are attributed to their capacity to retain inorganic marine substances due to the characteristics of their cell surface polysaccharides [6,19,68]. The mineral fraction of some seaweeds accounts for up to 36% of dry matter [17]. Many of these essential minerals accumulate in seaweeds at much higher levels than in terrestrial foodstuffs. For example, there is more iron in an 8 g serving of dry *Palmaria palmata* than in 100 g of raw sirloin steak [19]. All of the essential minerals and trace elements needed for human nutrition are present in seaweeds [68], and so it should be regarded as a valuable functional food. For instance, the brown algae, *Undaria pinnatifida* and *Sargassum,* and the red algae, *Chondrus crispus* and *Gracilariopsis*, can be used as food supplements to help meet the recommended daily intake of some minerals (Na, K, Ca, Mg) and trace elements (Fe, Zn, Mn, Cu) [68,69]. Moreover, analysis of the mineral composition of *Ulva rigida* revealed balanced contents of Na and K (15.9 and 15.6 g/kg respectively, ratio near to 1), which, from a nutritional point of view, is of interest as intake of diets with a high Na/K ratio have been related to incidence of hypertension [13]. Additionally, seaweeds are one of the most important vegetable sources of calcium. Calcium content may be as high as 7% of the dry weight in macroalgae and up to 25–34% in the chalky seaweed, lithotamne. Thus, seaweed consumption may also be useful to those at risk of calcium deficiency, namely expectant mothers, adolescents and the elderly [17].

Other bioactive compounds are the photosynthetic pigments used by autotrophs to capture solar energy for photosynthesis [4]. As regards macroalgae, the main pigments are carotenoids and chlorophylls (Table 1). The carotenoid fucoxanthin has potential commercial value as it has been reported to be of use in treating obesity and reducing the risk of certain diseases, such as type 2 diabetes through its ability to promote the expression of the uncoupling protein, UCP1 [70]. In the food industry, chlorophylls are mainly used as natural colorants in foods and beverages [4]. However, chlorophylls and their derivatives have been shown to possess some biological activity whereby they exhibit anticancer properties in their ability to bind carcinogenic hydrophobic compounds such as polycyclic aromatic hydrocarbons, heterocyclic amines and aflatoxin [71,72]. Phlorotannins, a group of polyphenolic compounds which have also been identified in several brown algal families, have been reported to possess strong antioxidant activity. However, at present, the extractable polyphenol levels from algae are lower than that of other phytochemicals [6,73,74].

The nutritional value ascribed to macroalgae along with their non-animal nature makes them particularly appropriate for use in the food industry. Seaweeds have enormous potential as components of fertilizers, in animal feed supplements, and as additives for human food. Hence, biotechnological advances regarding macroalgae cultivation has stimulated the development of seaweed aquaculture. At present, three genera, *Laminaria, Undaria* and *Porphyra*, constitute 93% of the algal mass cultivated for nutritional purposes [6].

### 2.2. Microalgae

Microalgae are the most primitive and simply organised members of the plant kingdom, with the majority existing as small cells of about 3–20 μm [4]. These algae are ubiquitous in nature and aquatic microalgae have been isolated in areas ranging from hot springs to glacial ice flows [75]. Microalgae are found in both benthic and littoral habitats and also throughout the ocean waters as phytoplankton. Phytoplankton comprises organisms such as diatoms (bacillariophyta), dinoflagellates (dinophyta), green and yellow-brown flagellates (chlorophyta; prasinophyta; prymnesiophyta, cryptophyta, chrysophyta and rhaphidiophyta) and blue-green algae (cyanophyta). As photosynthetic organisms, this group plays a key role in the productivity of oceans and constitutes the basis of the marine food chain [7].

There are over 50,000 different species of microalgae of which only a few have been characterised [75]. This group of microorganisms is extremely diverse and represents a major untapped resource of valuable bioactive compounds and biochemicals such as pigments, antioxidants, polysaccharides, sterols, fatty acids and vitamins [76].

#### 2.2.1. Proteins, Peptides and Amino Acids

The high protein content of various microalgal species and their amino acid pattern, which compares favourably with that of other food proteins, is a good endorsement of microalgae as an alternative protein source [77,78]. *Spirulina*, for instance, is high in protein (60–70% depending on the strain) and, not only does this protein possess all of the essential amino acids, but these amino acids have excellent bioavailability [52]. Furthermore, the industrial scale growth of the microalga, *Dunaliella*, can turn out protein extract at about 100 times greater productivity than that reported in agriculture and 50 fold greater than in fish farming [4].

Proteins from marine sources show promise as functional ingredients in foods because they possess numerous important and unique properties such as film and foaming capacity, gel forming ability and antimicrobial activity [4]. In addition, purified peptides from *Chlorella vulgaris* have demonstrated significant protective effects against cellular damage [79]. With regard to one of the major proteins in *Spirulina platensis* and *Porphyridium*, phycobiliprotein, several therapeutic bioactivities have been described, namely, hepatoprotective, anti-inflammatory, immunomodulating, antioxidant and anticancer effects [52].

#### 2.2.2. Fatty Acids

The average lipid content of algal cells varies between 1 and 70% but can reach 90% of dry weight under certain conditions [80]. Algal lipids are composed of glycerol, sugars or bases esterified to saturated or unsaturated fatty acids. Among all the fatty acids in microalgae, some fatty acids of the n-3 and n-6 families are of particular interest [42]. According to Mendes *et al.* [60], the main constituents of the lipidic fractions of *Chlorella vulgaris* are oleic, palmitic and linolenic acids, accounting for 41, 22 and 9% of the total amount, respectively. Additionally, palmitic, linolenic and oleic acids account for more than 85% of the total fatty acid content of *Dunaliella salina* [81], while the green microalga, *Haematococcus,* has been shown to contain short chain fatty acids with antimicrobial activity [66].

Higher plants and animals lack the requisite enzymes to synthesize PUFAs of more than 18 carbons and so have to obtain them from their food. Fish and fish oil are the common sources of long chain PUFAs but safety issues have been raised because of the possible accumulation of toxins in fish. Moreover, the application of fish oil as a food additive is limited due to problems associated with its typical fishy smell, unpleasant taste and poor oxidative stability [78]. Consequently, long chain PUFAs are commercially produced via microalgae cultivation for incorporation into infant milk formulations and for use as dietary supplements and food additives [78]. Maximum n-3 fatty acid production can also be induced by altering the growth conditions of microalgae. For instance, under optimal culture conditions, *Chlorella minutissima* can produce an EPA content of up to 45% of its total fatty acid content [4].

Microalgae such as *Porphyridium,* which shows a relatively low lipid content, contains significant amounts of several major fatty acids such as palmitic acid, AA, EPA and linoleic acid [46,52]. *Spirulina* provides an interesting source of γ-linolenic acid (20–25% of the total lipid fraction), which is a precursor of prostaglandins, leukotrienes and thromboxans involved in the modulation of immunological, inflammatory and cardiovascular responses [17]. This microalga is also a natural source of active fatty acids such as lauric, palmitic and oleic acids [82], with the n-3 fatty acid, docosahexaenoic acid (DHA), accounting for up to 9.1% of the total fatty acids content. *Spirulina* has been found to contain sterols, including clionasterol which has been shown to increase the production of plaminogen-activating factor in vascular endothelial cells [76].

#### 2.2.3. Polysaccharides

Carbohydrates in microalgae can be found in the form of starch, glucose, sugars and other polysaccharides. Their overall digestibility is high, which is why there is no limitation to using dried whole microalgae in foods or feeds [78]. Moreover, the biological activities of some microalgal species have been associated with polysaccharides. Polysaccharide complexes from *Chlorella pyrenoidosa*, and possibly *Chlorella ellipsoidea*, contain glucose and any combination of galactose, rhamnose, mannose, arabinose, *N*-acetylglucosamide and *N*-acetylgalactosamine. These complexes are believed to have immunostimulating properties, specifically immune stimulatory activity and can inhibit the proliferation of *Listeria monocytogenes* and *Candida albicans* [76]. The most important substance in *Chlorella* is β-1,3-glucan, which is an active immunostimulator, a free radical scavenger and a reducer of blood lipids. *Chlorella* can also be used as a food additive owing to the taste and flavour adjusting actions of its colouring agent [78]. Also, novel polysaccharides isolated from *Porphyridium* and *Nostac flegelliforme* microalgae exhibited potent antiviral activity against herpes simplex virus (HSV-1 and 2) both *in vitro* and *in vivo* [83,84].

#### 2.2.4. Antioxidants

The nutritional and therapeutic relevance of dietary carotenoids is attributed to their ability to act as provitamin A; that is, they can be converted into vitamin A by the human body. Moreover, carotenoids play a protective role by preventing the formation of reactive oxygen species [85]. Microalgal production of carotenoids, such as β-carotene and astaxanthin, is an attractive area of research as they are valuable bioactive ingredients that can present at relatively high concentrations in algal cells (Table 1). Moreover, cultivated algae can be induced to produce even larger quantities of carotenoids by controlling certain environmental growth conditions. The strains of microalgae that are currently being investigated for use as natural producers of commercial carotenoids include *Dunaliella salina, Sarcina maxima, Chlorella protothecoides, Chlorella vulgaris* and *Haematococcus pluvialis* [4].

*Dunaliella salina* is the most suitable organism for the mass production of β-carotene as it can produce β-carotene up to 14% of its dry weight [80]. This microalga can also be cultivated easily and quickly when compared to plants and produces both *cis* and *trans* isomers of carotenoids for high bioavailability and bioefficacy [85,86]. β-carotene is one of the leading food colorants in the world and has been applied to a range of food and beverage products to improve their appearance to consumers [87]. In addition, β-carotene has strong antioxidant properties which help to mediate the harmful effects of free radicals implicated in numerous life-threatening diseases, including various forms of cancer, coronary heart disease (CHD), premature aging and arthritis. The antioxidant qualities of β-carotene can also assist the body in suppressing the effects of premature aging caused by UV rays [28,86].

Microalgal-derived β-carotene has been reported to be more biologically active than synthetically produced β-carotene and can be marked as a “natural” food additive [4]. Natural β-carotene also contains numerous carotenoids and essential nutrients that are not present in the synthetic form and can be consumed in larger quantities as the body tissues regulate its use [88]. Additionally it has been observed that, under irradiance stress, *Dunaliella salina* can accumulate significant amounts of xanthophylls, particularly zeaxanthin, which possess unique biological properties with potential for disease prevention [53].

*Haematococcus* is another unicellular alga that can be used in both open and closed culture systems for the production of antioxidants, namely chlorophylls and carotenoids [4]. Under stress conditions, *Haematococcus pluvialis* has the ability to accumulate large quantities (1.5–3% of dry weight) of the high value carotenoid, astaxanthin [62]. Besides, the United States Food and Drug Administration for marketing has cleared *Haematococcus pluvialis* as a dietary supplement and it has also been approved in several European countries for human consumption [76]. With an antioxidant activity up to 10 times stronger than other carotenoids, astaxanthin provides protective activity against cancer, inflammation and UV light. The health benefits of astaxanthin along with its strong colouring properties make it a potential ingredient for use in the nutraceutical, cosmetics, food and feed industries [89].

Microalgae also represent a valuable source of nearly all essential vitamins (A, B_1_, B_2_, B_6_, B_12_, C, E, nicotinate, biotin, folic acid and pantothenic acid) and are generally rich in chlorophylls (Table 1) [78].

It has clearly been established that microalgae are a rich source of nutritious and biologically active compounds, namely carotenoids, phycobilins, fatty acids, polysaccharides, vitamins and sterols. Nevertheless, not only is it their huge diversity that makes these microorganisms interesting, but also the possibility of growing them at different conditions and using them as natural reactors, leading to an enrichment of some bioactive compounds. However, prior to this, algal material must be analysed for the presence of toxic compounds.

Despite the growing promise of microalgae as a source of food ingredients, the industry has developed with only varying amounts of success and its biotechnological potential remains to be fully exploited [4]. One such future application could be in the production of special lipids. The n-3 fatty acids found in the oils of certain cold water marine fish, which are believed to be capable of reducing the incidence of CHD, are likely to originate from the phytoplankton in food chain. Many of these phytoplankton species are found to be rich in reserves of oils containing various amounts of EPA and DHA [75]. Indeed, the oil obtained from the microalga *Schizochytium* sp. has been authorized by the United States to be used as a new food ingredient because of its high DHA (n-3) content and because it contains higher levels of squalene and phytosterols but three times less cholesterol than fish oil [6,78].

### 2.3. Byproducts of Processing

Byproducts of processing are generated when the fish/shellfish is gutted, headed and further processed either onboard fishing vessels or in processing plants on shore [90]. Production of marine-based food ingredients from these byproducts is a growing area of interest as it could help to reduce processing waste, thereby catering to ethical and environmental concerns over discards, and primarily, it could result in the development of valuable nutraceutical or functional food formulations [4]. According to Kelleher [91], discards from the world’s fisheries in 2005 exceeded 7 million tons, with only 50% of total catch being used for actual human consumption [92]. Fish heads, viscera, skin, tails, offal and blood, as well as seafood shells possess several compounds suitable for human health applications [93]. Studies have identified compounds from remaining fish muscle proteins, collagen and gelatin, fish oil, fish bone, internal organs, and shellfish and crustacean shells [94,95].

These bioactive compounds can be extracted and purified with various technologies leading to the preparation and isolation of bioactive peptides, oligosaccharides, fatty acids, enzymes, water soluble minerals and biopolymers for biotechnological and pharmaceutical applications [96].

#### 2.3.1. Proteins, Peptides and Amino Acids

Fish muscle proteins derived from processing byproducts can be hydrolysed enzymatically to recover protein biomass otherwise discarded as processing waste. Bioactive peptides isolated from various fish protein hydrolysates have shown numerous bioactivities such as antihypertensive, antithrombotic [96–98], anticoagulant [99], immunomodulatory and antioxidative activities [99,100]. Moreover, Jung *et al.* [101] reported that fish peptides are also capable of accelerating calcium absorption.

Some of the most prevalent marine proteins used in foods are collagen, gelatin and albumin, all of which can be extracted from fish and seafood byproducts [4]. Collagen and gelatin are unique proteins as they are rich in non-polar amino acids (above 80%) such as glycine, alanine, valine and proline [96]. Collagen is a connective tissue protein found in skin, bones, cartilage, and ligaments which can be extracted from fish processing byproducts [4]. Collagen derived from species living in warmer environments (e.g., tuna) have higher contents of proline and hydroxyproline, so they present a higher melting point and superior thermal stability than those from fish that live in cooler environments (e.g., cod) [93]. Gelatin is a protein product formed by the partial hydrolysis of collagen. It has a unique gel forming ability [4] and is used as a food additive to increase the texture, the water holding capacity and stability of several food products [92]. Traditionally, gelatin has been derived from beef or pork; however, marine gelatin can also be extracted from the skins of flatfish, cold water fish species or alternative sources such as squid and octopus [4,102]. Gelatin possesses a characteristic melt-in-the-mouth property, which makes it suitable to a wide range of applications in food and pharmaceutical industries; in particular, fish gelatin has a better release of aroma and shows a higher digestibility than animal gelatin [103].

Other bioactive proteins that can be obtained from marine processing include albumin and protamine. Albumin, has exhibited several properties that make it beneficial to human health, such as antioxidant and anticoagulatory activities and the ability to maintain microvascular integrity [104]. While it is typically derived from egg whites, albumin can also be isolated from mollusks, crustaceans and low fat fish [4]. Protamine is a simple peptide consisting largely of arginine residues that is found in the testicles of more than 50 fish species. Protamine is a promising antibacterial agent in food processing and preservation as it has the ability to prevent growth of Bacillus spores [92]. Also, major marine enzymes are produced as a result of fish and shellfish processing. These enzymes are valuable as food ingredients and in food processing due to their specificity, diverse properties, salt tolerance, and high activity at mild pH [4]. However, different opinions exist as to the cost and economy in extracting these enzymes as opposed to having them produced by microorganisms.

#### 2.3.2. Fatty Acids

Better utilization of marine fish processing byproducts could be achieved by converting these materials into fish oil [96]. The liver of lean white fish such as cod species, the muscle of fatty fish (herring, mackerel, salmon) and offal generated from processing are all good sources of marine lipids [92,96]. The fat content of fish varies from 2–30% and is mainly composed of two types of PUFAs, EPA and DHA [96]. Compared to saturated fats, PUFAs in fish oil are readily digested for energy production [96] and are believed to be the main protective components of fish oil that act against certain types of diseases.

Cod liver oil has long been used as a fish oil supplement as it contains high amounts of PUFAs, much of which is the n-3 fatty acid, EPA [105]. Although supplements are popular in Europe and Japan, a more attractive option for many in the food industry is to enrich everyday products like bread, egg, margarine *etc.* with n-3 long chain PUFAs [4]. However, the main factor limiting the application of these PUFAs in food products is their susceptibility to lipid oxidation, which can result in strong fishy odours and flavours [4,92].

#### 2.3.3. Polysaccharides

Chitin is ubiquitous in marine polysaccharides; it is one of the major structural components of crustacean shells and shellfish wastes with a structure similar to that of cellulose, and built from *n*-acetyl-glucosamine monomers [106]. On a dry weight basis, shrimp, crab, lobster, prawn and crayfish have been reported to contain between 14 and 35% chitin, while deproteinized dry shell waste of Antarctic krill contains approximately 40% crude chitin. As the insolubility of chitin hampers most of its applications, once isolated, chitin can be deacetylated to create chitosan, a large cationic polymer with numerous commercial applications in the food, pharmaceutical and waste treatment industries [4]. In practice, chitin is used almost exclusively as raw material for production of chitosan, oligosaccharides and glucosamine [93]. There are a variety of food applications for chitin, chitosan and their derivatives, including use as antimicrobial agents, edible films, additives, nutraceuticals (e.g., increasing dietary fibre, reducing lipid absorption) and water purifiers [107].

Chito-oligosaccharides are chitosan derivatives that can be generated via chemical or enzymatic hydrolysis of chitosan. Recently, these oligosaccharides have been the subject of increased attention in terms of their pharmaceutical and medicinal applications, due to lack of toxicity, high solubility and their positive physiological effects such as angiotensin-I-converting enzyme (ACE) inhibition, antioxidant, antimicrobial, anticancer, immunostimulant, hypocholesterolemic, hypoglycemic and anticoagulant properties [31].

#### 2.3.4. Calcium and Astaxanthin

Fish bone, which is separated after removal of muscle proteins on the frame, is a valuable source of calcium, which is an essential element for human health. As calcium is deficient in most regular diets, demand for calcium fortified products is growing continuously, and fish bone material is a useful source [108]. However, in order to incorporate fish bone into calcium fortified food it needs to be converted into an edible form by softening its structure [96].

Astaxanthin represents between 74 and 98% of the total pigments in shellfish. Due to these high contents, crustacean shells can not only be used for recovery of chitin but also for recovery of astaxanthin. Owing to its useful properties, astaxanthin from natural sources is increasingly being marketed as a functional food ingredient with prices ranging between $3,000 and $12,000 per kg. The methods currently available for the extraction of astaxanthin from shell matrices employ different elements such as edible oils, hydrochloric acid and organic solvents. Also, a feasible technique for partial concentration of astaxanthin from crustacean shells is via lactic acid fermentation, which also has the advantage of protecting the biomass from bacterial decomposition. The silage formed contains insoluble chitin, a protein rich fraction, and a lipid rich fraction composed of astaxanthin, sterols, and vitamins A and E [93].

#### 2.4. Other Benthic Species

The majority of bioactive marine molecules have been isolated from benthic species such as sponges, bryozoans, echinoderms, polychaetes, ascidians, mollusks and cnidarians [109]. These molecules have recognized applications against cancer, inflammation, HIV-AIDS, thrombotic disorders and infectious diseases [110]. In fact, more ascidian- and sponge-derived compounds are in clinical and preclinical trials than compounds from any other marine taxa [109].

An emerging source of new bioactive ingredients may result from the microbial diversity in the marine environment, particularly those microbes associated with marine plants and animals. Several studies have demonstrated that “living surfaces” represent an environment rich in epibiotic microorganisms that produce bioactives [111]. Many of these marine microorganisms can be easily cultured and manipulated in bioreactors and, therefore, represent an excellent renewable source of biologically active compounds. Some deep sea bacteria have been found to contain large amounts of EPA and DHA, presumably to allow their membranes to be fluid and adaptive to extreme temperatures and pressures [4]. For example, *Mortierella alpina* can produce EPA as 15% of total extractable fatty acid at 12 °C [112]. Moreover, extremophiles contain polysaccharides with a wide variety of chemical and physical properties that are often not present in or are variations of the more traditional, terrestrial plant-derived polysaccharides. One strain of *Alteromonas* has been found to produce an anionic exopolysaccharide with potential use as a thickening agent, while other *Altermonas* strains produced polymers with qualities such as unusual gelling properties, significant thickening ability, and high metal binding capacity [113]. In addition, halophiles such as *Halobacterium mediterranei* have been reported to contain exopolysaccharides with highly favourable rheological properties and resistance to high salinities, temperatures and pH [4]. Other promising sources of functional food ingredients include a red coloured bacterium obtained from Puerto Rico which was found to excrete vitamin B and antibacterial substances into the sea water [75], while Dharmaraj *et al.* [114] confirmed the production of food grade carotenoids by *Streptomyces* microbes isolated from the marine sponge *Callyspongia diffusa*.

Lower invertebrates, such as sponges, represent a great diversity of lipid components, such as fatty acids, sterols, and other unsaponifiable compounds, as well as compounds such as bioactive terpenes, cyclic peptides, alkaloids, peroxides, and amino acid derivatives [115]. However, sponge mariculture has not yet proven to be very lucrative as little is known about how to replicate the sponge’s natural environment and life cycle. Also, the bioactive compounds of interest are often only produced in trace amounts [4,115].

Another prospective source of n-3 long chain PUFAs is the class of algae-like fungi called phycomycetes. These marine fungi have been reported to produce significant levels of γ-linolenic acid, AA, EPA and DHA [4].

## 3. Potential to Reduce Prevalence of Chronic Diseases

The increasing number of scientific papers published in the last two decades correlating diet and some chronic diseases have shown the extraordinary possibilities of foods to support, or even to improve, our health. As a consequence, there is now a huge interest among consumers and the food industry on products that can promote health and well-being [5]. The marine world represents a largely untapped reserve of bioactive ingredients and considerable potential exists for exploitation of these compounds as functional food ingredients (Table 2). Substances such as chitin, chitosan, n-3 oils, algae, carotenoids, vitamins and minerals, calcium in fish bone, bioactive peptides and fish protein hydrolysates provide a myriad of health benefits, including reduction of CHD, anticarinogenic and anti-inflammatory activities [4,8].

An important element of nutritional discovery is the molecular target or pathway selected for modulation. Nutritional bioactives aiming at the prevention of diseases or slowing disease progression will at least partly overlap with those targeted by the pharmaceutical industry such as enzymes, receptors, or transcription factors. Additionally, elements that maintain cell homeostasis upstream of the final events that lead to the pathophysiological deteriorations may also be targeted by nutritional compounds [116]. In proving a defined health effect, however, the health promoting effect of these naturally occurring bioactive substances must be preventive, fundamentally distinguishing them from curative drugs [1].

### 3.1. Cancer

Many potent natural products which display effective anticancer activities have been discovered in the marine environment. Indeed, since the early 1990s, there has been a dramatic increase in the number of preclinical anticancer lead compounds from marine sources that have entered into human clinical trials [139,140]. One of these compounds, trabectidin (Yondelis^®^), originally isolated from the Caribbean marine tunicate *Ecteinascidia* turbinate, has been approved for use as an anticancer agent in Europe [141,142]. In addition, dehydrodidemnin B (aplidine), a compound extracted from the Mediterranean marine tunicate, *Aplidium albicans*, has been shown to be a powerful antitumor agent with possible applications in treating prostate, gastric, breast and colon cancers [140,143].

Marine-derived anticancer molecules have varying modes of action. A cell culture study by Russo *et al.* [144] reported that two lichen metabolites, sphaerophorin and pannarin, prevented UV light and nitric oxide mediated plasmid DNA damage, and attenuated the growth of melanoma cells by, at least in part, triggering an apoptotic process. Moreover, a number of isolated marine sponge compounds are inhibitors of protein kinase C (PKC) e.g., BRS1, isoaaptamine, debromohymenialdisine. PKC inhibitors have attracted much attention as there is evidence that too high levels of PKC enzyme are involved in the pathogenesis of arthritis and psoriasis, and in tumor development. PKC is believed to be the receptor protein of tumor promoting phorbol esters, and PKC inhibitors prevent binding of carcinosarcoma cells to the endothelium [145]. The cytoskeleton is also an interesting target for cancer therapy, as the microtubules and microfilaments are involved in cellular organisation during cell division. A number of extracts of marine sponges and ascidians are believed to inhibit the protein by binding to the microtubule binding site, “locking up” the protein’s motor function, and thereby blocking cell division [145–147]. Other metabolites can inhibit cell division by disrupting the polymerisation of actin [148,149], inhibition of cyclin-dependent kinase 4 [150,151], inhibition of protein synthesis [152,153] or by blocking topoisomerase II [154–156], the nuclear enzyme which makes transient DNA breaks that are required for replication [157].

In contrast, incubation with kahalalide F, a cyclic depsipeptide from the herbivorous marine mollusk, *Elysia rufescens*, quickly induced loss of mitochondrial membrane potential and lysosomal integrity, severe cytoplasmic swelling and vacuolisation, irregular clumping of chromatin within the cell nucleus, and finally, cell death in human cancer cells. These effects were independent of caspase activation and were not associated with DNA degradation or cell cycle block. Kahalalide F has shown to be effective against cell lines with strong multidrug resistance and against cell lines resistant to topoisomerase II inhibitors. *In vivo* models have also confirmed activity in various solid tumor models [158,159]. To date, copious numbers of compounds with reported anticancer activities have been extracted from marine organisms, however, for some, their exact effects are still unclear [140,142,145].

#### 3.1.1. Algal Polysaccharides

Wijesekara *et al.* [129] recently reported that algal sulfated polysaccharides have potent capacities for new anticancer product developments in the pharmaceutical as well as the food industries. Indeed, several *in vivo* mouse studies have demonstrated the antitumor activity of marine-based polysaccharides [94,160–164]. Moreover, non-digestible oligosaccharides, which are found in abundance in macroalgae, are known to improve gut microecology and in doing so may reduce the risk of colon cancer. In a review by Mussatto and Mancilha [38], the authors state that intake of transgalactosylated disaccharides reduces the faecal pH as well as ammonia, *p*-cresol and indole concentrations, with an increase in bifidobacteria and lactobacilli and a decrease in *Bacteroidaceae* populations. As these changes in faecal physiological parameters are believed to reduce the risk of cancer development, macroalgal non-digestible oligosaccharides could be considered as potential anticarcinogenic food ingredients [34,38].

#### 3.1.2. n-3 Polyunsaturated Fatty Acids

While the cardioprotective effects of fish oil n-3 PUFAs are well established, their antitumoral effects are not widely acknowledged. However, promising data from experimental studies carried out in animals show that elevated supplies of EPA, DHA and/or fish oil diet supplementation generally inhibit tumor growth and metastases occurrence [121,122,165–167]. Using a mouse model of MDA-MB-231 human breast cancer cell metastasis to bone, research by Mandal *et al.* [120] found that a fish oil diet enriched in DHA and EPA prevented the formation of osteolytic lesions in bone, indicating suppression of cancer cell metastasis to bone. The study also revealed markedly reduced levels of CD44 mRNA and protein (associated with generation of cancer stem cells) in the tumors of mice fed fish oil diet compared to those fed the control diet. Furthermore, Brown and colleagues [168] provided *in vitro* evidence supporting epidemiological data that the dietary ratio of n-3:n-6 is crucial in determining the risk of metastatic disease in prostate cancer. Increasing the ratio of n-3 to n-6 PUFAs, in particular increasing the amount of EPA in the diet, can inhibit the metastatic process by blocking the production of prostaglandin E2 and thereby reducing the risk of aggressive disease.

#### 3.1.3. Carotenoids and Chlorophylls

Being lipid soluble, carotenoids are absorbed with fats and circulate bound to different lipoproteins. The principal biological effects of carotenoids relate to their antioxidant properties, which form the basis of potential protection against lipid peroxidation, atherogenesis, DNA oxidation, and cancer [169]. Carotenoids have been implicated in the inhibition of cancer cells *in vitro* [170–172], in animal models [173,174] and in humans, as important dietary phytonutrients having cancer preventive activity for lung, colon, breast and prostate cancer [175,176]. As regards marine-sourced carotenoids, Cha *et al.* [134] found that the carotenoid extract of *Chlorella ellipsoidea* exerted strong antiproliferative effects on human colon cancer cells, including induction of apoptosis. The authors suggest that bioactive xanthophylls of *C. ellipsoidea* could be potential therapeutic agents in the prevention of human cancers. Several studies have also demonstrated the anticancer activity of astaxanthin in mammals [177–179].

The cancer preventative effect of chlorophyll derivatives have been extensively studied, with particular emphasis on their *in vitro* antimutagenic activity against numerous dietary and environmental mutagens [180–184]. In a study presented by Chernomorsky *et al.* [71], the authors conclude that food sources that yield chlorophyll derivatives may play a significant role in cancer prevention. They found that dietary chlorophyll derivatives exhibit antimutagenic effects and reduce tumor cell growth. Indeed, epidemiological evidence has linked diets high in chlorophyll with a reduced risk of colon cancer in humans [185]. Antioxidant activity, mutagen trapping, modulation of detoxification pathways, and induction of apoptosis in cancer cells have been highlighted as possible modes of actions responsible for chlorophyll’s protective effects *in vivo* [186].

Neither carotenoids nor chlorophylls can be synthesized by animal tissues [187]. Thus, these molecules must be obtained from food and, as previously illustrated, the marine environment represents an endless resource. Carotenoid and chlorophyll molecules can be extracted and used as natural colorants and antioxidants to restore the natural level of these molecules in food or to prepare fortified products. They can also be chemically modified before being incorporated into food products [187]. Overall, positive data from *in vitro* and animal studies have prompted an increased interest in the potential usefulness of carotenoids and chlorophylls as preventative agents for human populations at elevated risk of development of specific cancers [186].

### 3.2. Cardiovascular Disease

Cardiovascular disease (CVD) is a class of diseases that affect the heart, blood vessels (arteries and veins) and blood circulation, and is one of the leading causes of mortality and morbidity worldwide. Examples of CVD include atherosclerosis, CHD, stroke, heart failure, deep vein thrombosis and peripheral arterial disease. In relation to marine bioactives, there is considerable evidence that these compounds can help to reduce the risk factors associated with CVD. Low density lipoprotein (LDL) cholesterol was significantly lower in rats fed a diet containing dried *Ulva rigida* [13], while Oben and colleagues [188] found that individuals given a freshwater algae infusion displayed lower total cholesterol, LDL cholesterol and triglyceride levels, and higher high density lipoprotein (HDL) cholesterol values than those given a water placebo.

#### 3.2.1. Polysaccharides

Numerous epidemiological studies have shown a strong correlation between high fibre diets and a lower incidence of chronic disorders such as CVD [189–193]. Soluble fibre forms a viscous indigestible mass in the gut and helps trap digestive enzymes, cholesterol, starch, glucose, and toxins which are then expelled through the faeces. The soluble fraction of fibres has a hypocholesterolemic effect, possibly due to augmented gastrointestinal content interfering with micelle formation and lipid absorption, or an increase and excretion of neutral sterols and biliary acids. Given that seaweed contain a large amount of soluble polysaccharides, they therefore have potential function as dietary fibre [194]. Additionally, several investigators have reported that water soluble fractions of seaweeds or isolated algal polysaccharides induce hypocholesterolemic and antihypertensive effects in experimental animals [131,195,196]. Indeed, sodium alginate and fucoidan were found to decrease serum cholesterol levels *in vivo* [132,133,197].

#### 3.2.2. n-3 Polyunsaturated Fatty Acids

The amount of fat in the diet and the type of fatty acids consumed can influence the likelihood of CVD and its risk factors [22]. The first recognition of the beneficial effect of fatty acids on CVD came from the observations on the longevity of Eskimos, which was later attributed to the high contents of fish-derived n-3 long chain fatty acids (e.g., EPA and DHA) in their diets [198–201]. Since then, the cardioprotective effects of fish oil n-3 PUFAs appear well determined. Numerous epidemiological and experimental studies have conclusively shown that diets rich in fish and fish oils are associated with a reduced risk of CVD [202–211]. The omega-3 index, a proposed biomarker for CVD risk, is the level of EPA and DHA in red blood cell membranes (expressed as a percent of total fatty acids), and an index of 8% or greater has been posed as a target for reducing CVD [212]. Research carried out by Lerman *et al*. [213] found that provision of 2–3 g/day of EPA and DHA for 12 weeks increased the omega-3 index by 3.7 to 4.1 percentage points to cardioprotective levels. Moreover, fish oil supplementation has been shown to be beneficial in controlling high blood pressure [214–216].

#### 3.2.3. ACE-Inhibitory Peptides

High blood pressure (hypertension) is one of the major independent risk factors for CVD [217]. ACE plays a crucial role in the regulation of blood pressure [15], and so ACE inhibition is considered to be a useful therapeutic approach in the treatment of hypertension. Numerous investigations of marine-derived peptides have revealed potent antihypertensive and ACE inhibitory activities in hypertensive rats [218–221]. According to Lee *et al.* [222], a single oral administration of a peptide derived from tuna frame protein hydrolysate showed a strong suppressive effect on systolic blood pressure of spontaneously hypertensive rats and this antihypertensive activity was similar to that of captopril, a commercial antihypertensive drug. Therefore, due to their effectiveness in both prevention and treatment of hypertension, marine-derived bioactive peptides have prospective use as functional ingredients [15].

#### 3.2.4. Astaxanthin

Due to their antioxidant properties, carotenoids are believed to have therapeutic benefit in treating CVD [223,224]. In addition, astaxanthin, a carotenoid ubiquitous in the marine environment, exhibits anti-atherogenic effects. In a study involving hyperlipidemic rabbits, astaxanthin significantly reduced macrophage infiltration in lesions and lowered the occurrence of macrophage apoptosis and plaque ruptures [225]. Indeed, results of human intervention trials indicate that consumption of natural astaxanthin could contribute to prevention of atherosclerosis. Iwamoto *et al.* [226] reported a dose response relationship between astaxanthin and LDL oxidation time, while Yoshida and colleagues [227] recently demonstrated that astaxanthin intake ameliorates triglyceride and HDL cholesterol in correlation with increased adiponectin. Antihypertensive effects were also revealed when oral administration of astaxanthin for 14 days induced a significant reduction in arterial blood pressure in spontaneously hypertensive rats [228]. Furthermore, in a follow-up study, the authors suggest that astaxanthin could modulate the oxidative condition and may improve vascular elastin and arterial wall thickness in hypertension [229].

**Figure 1:**
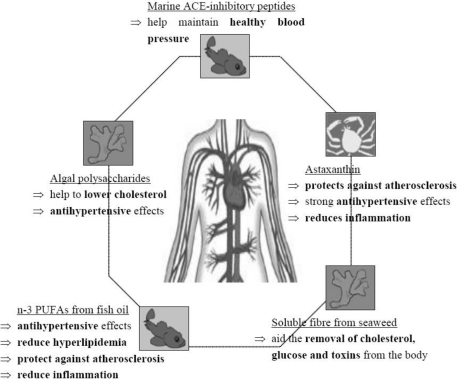
Beneficial effects of marine bioactives on the human cardiovascular system.

As diet is now recognised as an important modifiable risk factor for CVD, the incorporation of marine bioactives in food could benefit heart health by modifying blood levels of HDL and LDL cholesterol, as well as reducing hypertension (Figure 1). In particular, n-3 PUFAs and astaxanthin have use as dietary supplements for the prevention or the alleviation of certain CVD as they also reduce inflammation often associated with the development of CHD. Besides, several comprehensive reviews examine the literature on pharmacological studies of marine natural compounds that affect the cardiovascular system [145,230,231].

### 3.3. Inflammatory Conditions

Inflammation is a normal protective response to tissue damage or infection. However, if the response is exaggerated, misdirected or long term, inflammation can adversely affect health and give rise to many conditions such as inflammatory bowel disease, arthritis and asthma [22,86]. Interestingly however, owing to the involvement of inflammatory mediators called eicosanoids, a number of inflammatory conditions could potentially be alleviated by dietary modification [22].

As well as the various benefits accrued from the consumption of n-3 PUFAs as discussed previously, the eicosanoids derived from n-3 fatty acids are considered to be less inflammatory or even anti-inflammatory compared to eicosanoids derived from n-6 fatty acids [20]. Research has shown that increasing the balance of n-3 fatty acids in the diet, and consequently favouring the production of EPA in the body, or by increasing the dietary intake of EPA and DHA through consumption, leads the body to a more anti-inflammatory environment [232]. As a result, the incidence or severity of many chronic inflammatory diseases may be reduced [20,22,233]. In Crohn’s disease (a chronic inflammatory disease of the alimentary tract) for example, relapse rates reduced substantially over a 12 month period in patients receiving a fish oil supplement [234].

Another anti-inflammatory compound found extensively in the marine environment is the carotenoid astaxanthin. The antioxidative properties of astaxanthin are believed to be linked to its ability to relieve inflammation [89,224]. Bennedsen *et al.* [235] found that treatment with a cell extract from the microalgae *Haematococcus pluvialis* containing astaxanthin reduced bacterial load and gastric inflammation in *Helicobacter pylori* infected mice. It has also been reported that astaxanthin significantly reduces the production of pro-inflammatory mediators and cytokines, namely nuclear factor-κB (NF-κB), tumour necrosis factor-α (TNF-α) and interleukin-6 (IL-6) [236,237], and suppresses T lymphocyte activation in asthma patients [238]. However, further studies are needed to fully elucidate the anti-inflammatory effects of astaxanthin.

#### 3.3.1. Arthritis

Arthritis describes a condition involving inflammation of the joints and is a disease affecting mostly the aged population. Preventing inflammation with its associated pain and reduced mobility symptoms is a primary requirement in arthritis treatment [194]. Due to the involvement of inflammatory eicosanoids in the aetiology of this disorder, diet may be a potential therapeutic agent [22], and so the importance of dietary PUFAs in the treatment of arthritis has been greatly investigated [239–241]. Meta-analysis of 17 randomised, controlled trials was conducted by Goldberg and Katz [233] to assess the analgesic effects of n-3 PUFAs in patients with rheumatoid arthritis or joint pain. Favourable outcomes such as reduced patient reported joint pain intensity, minutes of morning stiffness, number of painful and/or tender joints and reduced non-steroidal anti-inflammatory drugs consumption were reported following 3 to 4 months supplementation with n-3 PUFAs. In fact, the authors conclude that n-3 PUFA supplementation is an attractive adjunctive treatment for joint pain.

In addition to n-3 PUFAs, clinical investigations suggest that ingestion of collagen hydrolysates, which can be isolated from fish waste, reduces pain in patients suffering from osteoarthritis [242].

#### 3.3.2. Asthma

Fish oil or fish containing more than 2% fat has been found to have a reduced risk of airway hyperresponsiveness, and children who regularly eat fresh, oily fresh have a reduced risk of developing asthma than children who rarely eat fish [243,244]. Supplementation of diet with n-3 fatty acids also confirmed their benefit in the reduction of breathing difficulties and other symptoms, along with reduced drug doses required by asthma patients. During treatment, the increase in the content of n-3 fatty acids in cell membranes was reported to take place at the expense of AA resulting in the competitive inhibition of pro-inflammatory eicosanoid production and production of anti-inflammatory eicosanoids [194,245,246]. Moreover, a number of interventional studies have demonstrated improvement in asthmatic status following n-3 PUFA supplementation [247–249]. Emelyanov *et al.* [123] assessed the effect of n-3 PUFA rich lipid extract of New Zealand green lipped mussel on symptoms, peak expiratory flow and exhaled hydrogen peroxide (a marker of airway inflammation) in patients with atopic asthma. This double blind randomised trial revealed a significant decrease in daytime wheeze, reduction in the concentration of exhaled hydrogen peroxide and an increase in morning peak expiratory flow compared to the placebo group.

#### 3.3.3. Neuroinflammation

Recently, there has been recognition of an inflammatory component to the pathology of neurodegeneration, most notably in Alzheimer’s disease but also in Parkinson’s disease and motor neuron disease [250]. As anti-inflammatory n-3 PUFAs are preferentially incorporated in the brain, a diet rich in EPA and DHA could keep neuroinflammation at a minimum [251]. In fact, elderly people who eat fish or seafood, that are highly enriched in n-3 PUFAs, at least once a week, have been shown to be at lower risk of developing dementia including Alzheimer’s disease [252,253].

Evidence is also emerging which suggests that marine algae could possess therapeutic activities for combating neurodegenerative diseases associated with neuroinflammation. Jin and colleagues [254] found that *Ulva conglobata* extract almost completely suppressed the expression of the pro-inflammatory enzyme cyclooxygenase-2 (COX-2) and inducible nitric oxide (iNOS) in murine BV2 microglia. Similarly, the brown alga, *Ecklonia cava*, was reported to induce significant inhibition of NF-κB dependent cytokines as well as iNOS and COX-2, thus reducing inflammation [255]. In addition, red alga *Neorhodomela aculeate* could be considered as a potential neuroprotective and anti-inflammatory agent to treat aging related neurological disorders [256]. However, as highlighted by the authors, further studies are needed to determine which components of each of the algae contribute to the observed anti-inflammatory activities.

As well as the nutritional bioactives discussed, numerous anti-inflammatory compounds with potential pharmacological applications have been isolated from marine sources [7,145,230,231,257,258]. Nevertheless, n-3 PUFAs appear to be the most prominent and most promising anti-inflammatory agents. In spite of this, there have not been sufficient studies to warrant a dietary recommendation regarding the use of n-3 PUFAs in the management of inflammatory conditions, and so there is a need for more carefully designed and controlled clinical trials in the therapeutic applications of n-3 fatty acids [22]. There is, however, a potential complementary role between drug therapy and a diet rich in n-3 PUFAs. An increased intake of n-3 fatty acids may increase the efficacy of anti-inflammatory medications, and perhaps reduce the need for conventional drugs. This dietary choice could be achieved through the use of marine-sourced PUFAs as fortificants in margarine, dairy products (milk, yoghurt, cheese), breads and baked goods, meat-based and fish-based meals, for example.

### 3.4. Cognitive Decline and Depression

Cognitive impairment and dementia are frequent disorders among elderly persons and influence the individual’s ability to function independently. Due to the aging of the population, the prevalence of cognitive impairment and dementia are expected to increase [259]. Drugs currently used in the treatment of cognitive decline and dementia have a very limited therapeutic value, suggesting the necessity to potentially individualise new strategies able to prevent and to slow down the progression of predementia and dementia syndromes [260]. Numerous epidemiological studies on the association between diet and cognitive decline suggest a role of fatty acids intake in maintaining adequate cognitive functioning and possibly in preventing or delaying the onset of dementia, both of degenerative or vascular origin [261–263]. In particular, fatty fish and marine n-3 PUFA consumption was associated with a reduced risk [264]. Moreover, a diet enriched with the algae, *Chlorella*, reduced oxidative stress and significantly prevented the decline of cognitive ability in an age dependent dementia mouse model. The authors suggest that the prolonged consumption of *Chlorella* has the potential to prevent the progression of cognitive impairment [265].

Several hypotheses could explain the association between dietary unsaturated fatty acids and cognitive functioning, including mechanisms through the co-presence of antioxidant compounds in food groups rich in fatty acids, via atherosclerosis and thrombosis, inflammation, accumulation of amyloid β-peptide, or via an effect in maintaining the structural integrity of neuronal membranes [260]. It is also believed that DHA is of particular importance for brain function as it maintains an optimal state of neural membranes, enabling membrane fluidity and thickness, which in turn affects cell signalling [266,267].

In general, PUFAs of marine origin appear to be suitable candidates for functional food ingredients to relieve memory deficits associated with aging. Promising results have already been reported in young population samples. Supplementation with a fish-flour bread spread containing n-3 PUFAs, embedded in a natural food matrix, had a beneficial effect on learning and memory of children [267]. In addition, a study involving marine collagen peptide (MCP) derived from Chum Salmon skin demonstrated that MCP facilitates learning and memory in aged mice by reducing oxidative damage in the brain and increasing brain derived neurotrophic factor and PSD95 protein expression [268].

Several epidemiological- and dietary-based studies also suggest that the consumption of n-3 fatty acids is inversely correlated to the prevalence and severity of depression, while clinical studies have presented evidence relating to the benefits of n-3 compounds in the treatment of depressive disorders [269,270]. In some trials, supplementation with long chain n-3 PUFAs has been shown to improve mood in patients with major depression and bipolar disorder [271–274]. More recently, Venna *et al.* [275] revealed that chronic supplementation with n-3 PUFAs induced antidepressant-like effects in mice, while in a similar mouse study, natural products isolated from marine sponges were found to possess antidepressant properties[276].

### 3.5. Diabetes

Dietary management of diabetes involves maintaining both blood glucose and blood lipid concentrations at as near normal levels as possible to reduce the possibility of associated complications developing, e.g., CVD, diabetic neuropathy, retinopathy and nephropathy [22]. As well as offering protection against heart disease and cancer, some marine extracts have been associated with improvements in glycaemic control (outlined in Table 3). Consequently, these compounds could be exploited as potential functional food ingredients in an effort to prevent or diminish insulin resistance and diabetes.

## 4. Conclusions

With increased life expectancy, our diet will play a key role in sustaining human health. This is a challenge for the food industry as consumers not only demand tasty and convenient food, but also healthy, nutritious food [116]. Marine nutraceuticals are both a coherent and attractive option for the food industry as there are a multitude of functional food ingredients that can be derived from marine sources. It is also believed that, as people become increasingly aware of the association between diet and good health, the consumption of fishery products will most likely increase [8]. Moreover, in relation to marine algae, their content in proteins, carbohydrates, lipids, fibre, metabolites, *etc.* can be influenced by their growing parameters (water temperature, salinity, light and nutrients) [5]. This means that, from a biotechnological perspective, algae can be considered as natural bioreactors, able to provide different types of compounds at different quantities—an appealing attribute to the functional food industry.

Despite the vast possibilities for use of marine bioactives in food, more multidisciplinary research is needed. All aspects, including chemical composition, biotechnology, extraction, bioactivity, and toxicity should be considered. Also, to effectively transfer the research into a practical field in which marine bioactives can be used as functional food ingredients, the exact functional activity of the extracts/compounds should be determined [52]. This primary objective should be based on investigations *in vitro* or *ex vivo* in cellular lines or culture tissues, later in animal models and then corroborated in studies of observation or intervention in human clinical trials [5]. Finally, to be employed as ingredients in food products, different studies should be carried out to determine if their activity is maintained after manufacturing and cooking processes [52].

In conclusion, marine bioactives appear to fit the criteria for development as functional food ingredients. Firstly, they are widely available, with a guaranteed supply. Secondly, marine bioactives are naturally occurring compounds, and their isolation/extraction is relatively cost effective. Lastly, and probably most importantly, they are functional—their biological activities affect the pathogenesis of several diseases. Consequently, ongoing efforts should be made into the research and development of marine functional foods with prospect that, in the future, their consumption could lead to a reduction in the prevalence and severity of chronic diseases.

## Figures and Tables

**Table 1:** Algal sources of dietary antioxidants.

**Antioxidant**	**Algal species**	**Reported levels (μg/g dry wt)**	**Reference**
Vitamin C	*Ulva* sp.	94.20–1250	[13,19]
	*Monostroma undulatum*	1590–4550	[6]
	*Undaria pinnatifida*	1847.38	[19]
	*Ascophyllum nodosum*	81.75	[19]
	*Laminaria digitata*	355.25	[19]
	*Porphyra umbilicalis*	1610.63	[19]
	*Palmaria palmata*	690	[19]
	*Thalassiosira pseudonana*	1100	[42]
	*Chaetoceros muelleri*	16000	[42]
	*Gracilaria changgi*	285	[43]

Vitamin E	*Ulva rigida*	19.70	[13]
	*Ascophyllum nodosum*	3.63	[19]
	*Dunaliella tertiolecta*	200–500	[44]
	*Undaria pinnatifida*	145–174	[19]
	*Laminaria digitata*	34.38	[19]
	*Porphyra umbilicalis*	14.25	[19]
	*Palmaria palmata*	162	[19]
α-tocopherol	*Porphyridium cruentum*	55.2	[45]
	*Laminaria ochroleuca*	8.9 ± 2.1	[41]
	*Saccorhiza polychides*	5.7 ± 1.3	[41]
	*Himanthalia elongata*	12.0–33.3	[41]
	*Tetraselmis suecica*	190–1080	[44]
γ-tocopherol	*Porphyridium cruentum*	51.3	[45]

Carotenoids	*Porphyridium cruentum*	1020 ± 140	[46]
α-carotene	*Chlorella pyrenoidosa*	4232.50	[47]
	*Dunaliella salina*	2410–2690	[48]
β-carotene	*Ascophyllum nodosum*		[26]
	*Chlorella pyrenoidosa*	4314.3	[47]
	*Chlorella vulgaris*	80–500	[49,50]
	*Chlorococcum*		[51]
	*Dunaliella salina*	4950–138250	[48,52–54]
	*Fucus serratus*		[26]
	*Fucus vesiculosus*		[26]
	*Gracilaria changgi*	52 ± 4	[43]
	*Haematococcus pluvialis*	80 ± 30	[55,56]
	*Laminaria digitata*		[26]
	*Laminaria saccharina*		[26]
	*Pelvetia canaliculata*		[26]
	*Phormidium* sp.		[57]
	*Porphyra tenera*		[58]
	*Synechocystis* sp.	2040	[59]
antheraxanthin	*Dunaliella salina*		[53]
	*Laminaria digitata*		[26]
	*Laminaria saccharina*		[26]
astaxanthin	*Chlorella vulgaris*		[60]
	*Chlorococcum* sp.		[51,61]
	*Haematococcus pluvialis*	up to 3%	[52,56,62]
β-cryptoxanthin	*Chlorella pyrenoidosa*	334.9	[47]
cantaxanthin	*Chlorella vulgaris*		[60]
	*Chlorococcum*		[51]
echinenone	*Phormidium* sp.		[57]
	*Synechocystis* sp.	240	[59]
fucoxanthin	*Ascophyllum nodosum*		[26]
	*Fucus serratus*		[26]
	*Fucus vesiculosus*		[26]
	*Hijikia fusiformis*		[27]
	*Himanthalia elongata*	820	[59]
	*Laminaria digitata*		[26]
	*Laminaria saccharina*		[26]
	*Pelvetia canaliculata*		[26]
loroxanthin	*Chlorella pyrenoidosa*		[63]
lutein	*Chlorella protothecoides*	4600	[64]
	*Chlorella pyrenoidosa*	1153009.70	[47,63]
	*Chlorella vulgaris*	2970–3830	[49,50]
	*Chlorella zofingiensis*	3400	[64]
	*Chlorococcum*		[51]
	*Dunaliella salina*	6550 ± 920	[48,53]
	*Haematococcus pluvialis*	270 ± 60	[55,56]
	*Muriellopsis* sp.	4300	[64]
	*Phormidium* sp.		[57]
	*Porphyra tenera*		[58]
	*Scenedesmus almeriensis*	4500	[64]
myxoxanthophyll	*Synechocystis* sp.	580	[59]
neoxanthin	*Ascophyllum nodosum*		[26]
	*Chlorella pyrenoidosa*	199.7	[47]
	*Dunaliella salina*		[53]
	*Fucus serratus*		[26]
	*Fucus vesiculosus*		[26]
	*Haematococcus pluvialis*	60 ± 20	[55,56]
	*Laminaria digitata*		[26]
	*Laminaria saccharina*		[26]
	*Pelvetia canaliculata*		[26]
	*Phormidium* sp.		[57]
violaxanthin	*Ascophyllum nodosum*		[26]
	*Chlorella pyrenoidosa*	38.1	[47,63]
	*Fucus serratus*		[26]
	*Fucus vesiculosus*		[26]
	*Haematococcus pluvialis*	40 ± 20	[55]
	*Himanthalia elongata*	50	[59]
	*Laminaria digitata*		[26]
	*Laminaria saccharina*		[26]
	*Pelvetia canaliculata*		[26]
	*Phormidium* sp.		[57]
zeaxanthin	*Ascophyllum nodosum*		[26]
	*Chlorella pyrenoidosa*	2170.3	[47]
	*Dunaliella salina*	11270 ± 1580	[48,53]
	*Fucus serratus*		[26]
	*Fucus vesiculosus*		[26]
	*Haematococcus pluvialis*	30 ± 10	[55]
	*Himanthalia elongata*	130	[59]
	*Laminaria digitata*		[26]
	*Laminaria saccharina*		[26]
	*Pelvetia canaliculata*		[26]
	*Synechocystis* sp.	1640	[59]

Chlorophylls	*Dunaliella salina*	26–3100	[53,65]
	*Himanthalia elongata*		[59]
chlorophyll *a*	*Chlorella pyrenoidosa*		[63]
	*Chlorella vulgaris*	3320–9630	[49,50]
	*Chlorococcum*		[51]
	*Phormidium* sp.		[57]
	*Porphyra tenera*		[58]
	*Porphyridium cruentum*	2130 ± 1200	[46]
	*Tetraselmis suecica*	6040–27530	[44]
chlorophyll *b*	*Chlorella pyrenoidosa*		[63]
	*Chlorella vulgaris*	2580–5770	[49,50]
	*Chlorococcum*		[51]
	*Haematococcus pluvialis*		[56]
	*Porphyridium cruentum*	380 ± 340	[46]
pheophytin *a*	*Chlorella vulgaris*		[50]
	*Porphyridium cruentum*	3310 ± 1110	[46]
pheophytin *b*	*Chlorella vulgaris*	2310–5640	[49,50]
	*Porphyridium cruentum*	30 ± 90	[46]

Polyphenols	*Fucus* sp.	41400 ± 400	[6]
	*Haematococcus pluvialis*		[66]
	*Laminaria* sp.	7300 ± 100	[6]
	*Porphyra* sp.	5700 ± 100	[6]
	*Spongiochloris spongiosa*	5.65	[67]
	*Undaria* sp.	6600 ± 100	[6]

**Table 2:** Potential marine functional food ingredients and their health benefits.

**Functional food ingredient**	**Health benefit**	**Marine source**	**Reference**
Peptides	ACE inhibition	Fish frame, algae	[15,31,97,98]
	Anticoagulative	Fish frame	[15,99]
	Antidiabetic	Fish frame	[117]
	Antimicrobial	Marine invertebrates, fish	[15,118]
	Antioxidative	Algae protein waste, fish frame	[15,79,95]

n-3 fatty acids	Anticarcinogenic	Fish	[119–122]
	Anti-inflammatory	Fish, mussels	[20,123]
	Cardioprotective	Fish	[124,125]
	Cognitive function	Fish	[126,127]

Polysaccharides	Anticarcinogenic	Algae, crustaceans (chito-oligosaccharides)	[94,128,129]
	Antioxidative	Algae, crustaceans (chito-oligosaccharides)	[129,130]
	Antiviral	Algae	[83,129]
	Cardioprotective	Algae	[131–133]

Carotenoids	Anticarcinogenic	Algae	[58,134]
	Antioxidative	Algae	[27,48]
	Anti-obesity	Algae	[70]
	Antidiabetic	Algae	[135]

Chlorophyll	Anticarcinogenic	Algae	[58,71]

Polyphenols	Antidiabetic	Algae	[136–138]
	Antioxidative	Brown algae	[73]

**Table 3:** Antidiabetic properties of marine natural products with established mechanisms of action.

**Compound**	**Source**	**Experimental model**	**Effect/Mechanism of action**	**Reference**
α-galactosylceramide	*Agelas mauritianus* sponge	Non-obese diabetic mice	Suppression of IFN-γ, increase of serum Ig E levels, and promotion of islet autoantigen specific Th2 cells Suppression of T- and B-cell autoimmunity to islet beta cells	[277,278]
Aqueous extracts	*Xetospongia muta* sponge, *Bunodosoma granulifera* and *Bartholomea annulata* sea anemones	*In vitro* models	Inhibition of dipeptidyl peptidase IV activity	[279]
Ethanolic extract	*Ulva rigida* alga	Wistar diabetic rats	Decreased blood glucose concentrations	[137]
Extract	*Posidonia oceanica* phanerogam	Wistar diabetic rats	Decreased blood glucose concentrations	[280]
Fucosterol	*Pelvetia siliquosa* alga	Sprague-Dawley diabetic rats	Reduction in serum glucose concentration and inhibition of sorbitol accumulation in the lenses	[281]
Marine collagen peptides	Wild fish	Human diabetic subjects	Decreased free fatty acids, cytochrome P450 and hs-CRP Regulation on metabolic nuclear receptors	[117]
Methanolic extract	*Ecklonia cava* alga	Sprague-Dawley diabetic rats	Reduction in plasma glucose levels and increased insulin concentration Activation of AMPK/ACC and PI3/Akt signalling pathways	[138]
Microalgal extracts	*Chlorella* sp. alga, *Nitzschia laevis* diatom	*In vitro* models	Inhibition of advanced glycation endproducts (AGEs) formation	[135]
n-3 PUFAs	Fish oil	Wistar rats	Restoration of insulin receptor and insulin receptor substrate-1 tyrosine phosphorylation Maintenance of phosphatidylinositol-3’ kinase activity and GLUT-4 content in muscle	[282]
	Fish oil	Healthy human subjects	Reduction in glucose oxidation, increased fat oxidation and glycogen storage	[283]
Phenylmethylene hydantoins	*Hemimycale arabica* sponge	*In vitro* model Sprague-Dawley rats	Inhibition of glycogen synthase kinase-3β activity Increased liver glycogen	[284]
Phlorotannin components	*Ascophyllum nodosum* alga	*In vitro* models	Inhibition of α-amylase and α-glucosidase activities	[136]
Sodium alginate	*Laminaria angustata* alga	Wistar rats	Inhibition of rising blood glucose and insulin levels	[197]

## References

[1] Biesalski H-K, Dragsted LO, Elmadfa I, Grossklaus R, Müller M, Schrenk D, Walter P, Weber P (2009). Bioactive compounds: Definition and assessment of activity. Nutrition.

[2] Honkanen P, Luten J (2009). Consumer acceptance of (marine) functional food. Marine Functional Food.

[3] Siró I, Kápolna E, Kápolna B, Lugasi A (2008). Functional food. Product development, marketing and consumer acceptance-a review. Appetite.

[4] Rasmussen RS, Morrissey MT (2007). Marine biotechnology for production of food ingredients. Adv Food Nutr Res.

[5] Plaza M, Cifuentes A, Ibáñez E (2008). In the search of new functional food ingredients from algae. Trends Food Sci Technol.

[6] Bocanegra A, Bastida S, Benedí J, Ródenas S, Sánchez-Muniz FJ (2009). Characteristics and nutritional and cardiovascular-health properties of seaweeds. J Med Food.

[7] El Gamal AA (2010). Biological importance of marine algae. Saudi Pharm J.

[8] Kadam S, Prabhasankar P (2010). Marine foods as functional ingredients in bakery and pasta products. Food Res Int.

[9] Mabeau S, Fleurence J (1993). Seaweed in food products: Biochemical and nutritional aspects. Trends Food Sci Technol.

[10] Fleurence J (1999). Seaweed proteins: Biochemical, nutritional aspects and potential uses. Trends Food Sci Technol.

[11] Dawczynski C, Schubert R, Jahreis G (2007). Amino acids, fatty acids, and dietary fibre in edible seaweed products. Food Chem.

[12] Galland-Irmouli A-V, Fleurence J, Lamghari R, Luçon M, Rouxel C, Barbaroux O, Bronowicki J-P, Villaume C, Guéant J-L (1999). Nutritional value of proteins from edible seaweed palmaria palmata (dulse). J Nutr Biochem.

[13] Taboada C, Millán R, Míguez I (2010). Composition, nutritional aspects and effect on serum parameters of marine algae *ulva rigida*. J Sci Food Agric.

[14] Pihlanto-Leppälä A (2000). Bioactive peptides derived from bovine whey proteins: Opioid and ace-inhibitory peptides. Trends Food Sci Technol.

[15] Kim S-K, Wijesekara I (2010). Development and biological activities of marine-derived bioactive peptides: A review. J Funct Foods.

[16] Elias RJ, Kellerby SS, Decker EA (2008). Antioxidant activity of proteins and peptides. Crit Rev Food Sci Nutr.

[17] Burtin P (2003). Nutritional value of seaweeds. EJEAFChe.

[18] Aneiros A, Garateix A (2004). Bioactive peptides from marine sources: Pharmacological properties and isolation procedures. J Chromatogr B.

[19] MacArtain P, Gill CIR, Brooks M, Campbell R, Rowland IR (2007). Nutritional value of edible seaweeds. Nutr Rev.

[20] Wall R, Ross RP, Fitzgerald GF, Stanton C (2010). Fatty acids from fish: The anti-inflammatory potential of long-chain omega-3 fatty acids. Nutr Rev.

[21] Calder PC (2006). n-3 Polyunsaturated fatty acids, inflammation, and inflammatory diseases. Am J Clin Nutr.

[22] Lunn J, Theobald H (2006). The health effects of dietary unsaturated fatty acids. Nutr Bull.

[23] Dembitsky VM, Pechenkina-Shubina EE, Rozentsvet OA (1991). Glycolipids and fatty acids of some seaweeds and marine grasses from the black sea. Phytochemistry.

[24] Sánchez-Machado D, López-Cervantes J, López-Hernández J, Paseiro-Losada P (2004). Fatty acids, total lipid, protein and ash contents of processed edible seaweeds. Food Chem.

[25] Piovetti L, Deffo P, Valls R, Peiffer G (1991). Determination of sterols and diterpenoids from brown algae (cystoseiraceae). J Chromatogr A.

[26] Haugan JA, Liaaen-Jensen S (1994). Algal carotenoids 54. Carotenoids of brown algae (phaeophyceae). Biochem Syst Ecol.

[27] Yan X, Chuda Y, Suzuki M, Nagata T (1999). Fucoxanthin as the major antioxidant in *hijikia fusiformis,* a common edible seaweed. Biosci Biotechnol Biochem.

[28] Dembitsky VM, Maoka T (2007). Allenic and cumulenic lipids. Prog Lipid Res.

[29] Gómez-Ordóñez E, Jiménez-Escrig A, Rupérez P (2010). Dietary fibre and physicochemical properties of several edible seaweeds from the northwestern spanish coast. Food Res Int.

[30] Jiménez-Escrig A, Sánchez-Muniz F (2000). Dietary fibre from edible seaweeds: Chemical structure, physicochemical properties and effects on cholesterol metabolism. Nutr Res.

[31] Wijesekara I, Kim SK (2010). Angiotension-i-converting enzyme (ace) inhibitors from marine resources: Prospects in the pharmaceutical industry. Mar Drugs.

[32] Berteau O, Mulloy B (2003). Sulfated fucans, fresh perspectives: Structures, functions, and biological properties of sulfated fucans and an overview of enzymes active towards this class of polysaccharide. Glycobiology.

[33] Pomin VH, Mourão PAS (2008). Structure, biology, evolution, and medical importance of sulfated fucans and galactans. Glycobiology.

[34] O’Sullivan L, Murphy B, McLoughlin P, Duggan P, Lawlor PG, Hughes H, Gardiner GE (2010). Prebiotics from marine macroalgae for human and animal health applications. Mar Drugs.

[35] Devillé C, Damas J, Forget P, Dandrifosse G, Peulen O (2004). Laminarin in the dietary fibre concept. J Sci Food Agric.

[36] Devillé C, Gharbi M, Dandrifosse G, Peulen O (2007). Study on the effects of laminarin, a polysaccharide from seaweed, on gut characteristics. J Sci Food Agric.

[37] Courtois J (2009). Oligosaccharides from land plants and algae: Production and applications in therapeutics and biotechnology. Curr Opin Microbiol.

[38] Mussatto SI, Mancilha IM (2007). Non-digestible oligosaccharides: A review. Carbohydr Polym.

[39] Wang Y (2009). Prebiotics: Present and future in food science and technology. Food Res Int.

[40] Rodríguez-Bernaldo de Quirós A, Castro de Ron C, López-Hernández J, Lage-Yusty M (2004). Determination of folates in seaweeds by high-performance liquid chromatography. J Chromatogr A.

[41] Sánchez-Machado D, López-Hernández J, Paseiro-Losada P (2002). High-performance liquid chromatographic determination of [alpha]-tocopherol in macroalgae. J Chromatogr A.

[42] Brown MR, Jeffrey SW, Volkman JK, Dunstan GA (1997). Nutritional properties of microalgae for mariculture. Aquaculture.

[43] Norziah MH, Ching CY (2000). Nutritional composition of edible seaweed *gracilaria changgi*. Food Chem.

[44] Carballo-Cárdenas EC, Tuan PM, Janssen M, Wijffels RH (2003). Vitamin e (α-tocopherol) production by the marine microalgae *dunaliella tertiolecta* and *tetraselmis suecica* in batch cultivation. Biomol Eng.

[45] Durmaz Y, Monteiro M, Bandarra N, Gökpinar Ş, Işik O (2007). The effect of low temperature on fatty acid composition and tocopherols of the red microalga *porphyridium cruentum*. J Appl Phycol.

[46] Rebolloso Fuentes MM, Acién Fernández G, Sánchez Pérez J, Guil Guerrero J (2000). Biomass nutrient profiles of the microalga porphyridium cruentum. Food Chem.

[47] Inbaraj BS, Chien JT, Chen BH (2006). Improved high performance liquid chromatographic method for determination of carotenoids in the microalga *chlorella pyrenoidosa*. J Chromatogr A.

[48] Hu C-C, Lin J-T, Lu F-J, Chou F-P, Yang D-J (2008). Determination of carotenoids in *dunaliella salina* cultivated in taiwan and antioxidant capacity of the algal carotenoid extract. Food Chem.

[49] Cha KH, Lee HJ, Koo SY, Song DG, Lee DU, Pan CH (2010). Optimization of pressurized liquid extraction of carotenoids and chlorophylls from *chlorella vulgaris*. J Agric Food Chem.

[50] Cha KH, Kang SW, Kim CY, Um BH, Na YR, Pan CH (2010). Effect of pressurized liquids on extraction of antioxidants from *chlorella vulgaris*. J Agric Food Chem.

[51] Yuan J-P, Chen F, Liu X, Li X-Z (2002). Carotenoid composition in the green microalga chlorococcum. Food Chem.

[52] Plaza M, Herrero M, Cifuentes A, Ibáñez E (2009). Innovative natural functional ingredients from microalgae. J Agric Food Chem.

[53] Yokthongwattana K, Savchenko T, Polle JEW, Melis A (2005). Isolation and characterization of a xanthophyll-rich fraction from the thylakoid membrane of *dunaliella salina* (green algae). Photochem Photobiol Sci.

[54] Herrero M, Jaime L, Martín-Alvarez PJ, Cifuentes A, Ibáñez E (2006). Optimization of the extraction of antioxidants from *dunaliella salina* microalga by pressurized liquids. J Agric Food Chem.

[55] Grewe C, Griehl C (2008). Time- and media-dependent secondary carotenoid accumulation in *haematococcus pluvialis*. Biotechnol J.

[56] Jaime L, Rodríguez-Meizoso I, Cifuentes A, Santoyo S, Suarez S, Ibáñez E, Señorans FJ (2010). Pressurized liquids as an alternative process to antioxidant carotenoids' extraction from *haematococcus pluvialis* microalgae. LWT-Food Sci Technol.

[57] Rodríguez-Meizoso I, Jaime L, Santoyo S, Cifuentes A, Garcia-Blairsy Reina G, Señoráns F, Ibáñez E (2008). Pressurized fluid extraction of bioactive compounds from *phormidium* species. J Agric Food Chem.

[58] Okai Y, Higashi-Okai K, Yano Y, Otani S (1996). Identification of antimutagenic substances in an extract of edible red alga, *porphyra tenera* (asadusa-nori). Cancer Lett.

[59] Plaza M, Santoyo S, Jaime L, García-Blairsy Reina G, Herrero M, Señoráns FJ, Ibáñez E (2010). Screening for bioactive compounds from algae. J Pharm Biomed Anal.

[60] Mendes RL, Fernandes HL, Coelho JP, Reis EC, Cabral JM, Novais JM, Palavra AF (1995). Supercritical co_2_ extraction of carotenoids and other lipids from chlorella vulgaris. Food Chem.

[61] Li H-B, Chen F (2001). Preparative isolation and purification of astaxanthin from the microalga chlorococcum sp. by high-speed counter-current chromatography. J Chromatogr A.

[62] Tripathi U, Sarada R, Rao SR, Ravishankar GA (1999). Production of astaxanthin in haematococcus pluvialis cultured in various media. Bioresour Technol.

[63] Wu Z, Wu S, Shi X (2007). Supercritical fluid extraction and determination of lutein in heterotrophically cultivated *chlorella pyrenoidosa*. J Food Process Eng.

[64] Del Campo JA, García-González M, Guerrero MG (2007). Outdoor cultivation of microalgae for carotenoid production: Current state and perspectives. Appl Microbiol Biotechnol.

[65] Macías-Sánchez MD, Mantell C, Rodríguez M, Martínez de la Ossa E, Lubián LM, Montero O (2009). Comparison of supercritical fluid and ultrasound-assisted extraction of carotenoids and chlorophyll a from *dunaliella salina*. Talanta.

[66] Rodríguez-Meizoso I, Jaime L, Santoyo S, Señoráns F, Cifuentes A, Ibáñez E (2010). Subcritical water extraction and characterization of bioactive compounds from *haematococcus pluvialis* microalga. J Pharm Biomed Anal.

[67] Klejdus B, Kopecký J, Benesová L, Vacek J (2009). Solid-phase/supercritical-fluid extraction for liquid chromatography of phenolic compounds in freshwater microalgae and selected cyanobacterial species. J Chromatogr A.

[68] Rupérez P (2002). Mineral content of edible marine seaweeds. Food Chem.

[69] García-Casal MN, Pereira AC, Leets I, Ramírez J, Quiroga MF (2007). High iron content and bioavailability in humans from four species of marine algae. J Nutr.

[70] Maeda H, Hosokawa M, Sashima T, Funayama K, Miyashita K (2005). Fucoxanthin from edible seaweed, *undaria pinnatifida,* shows antiobesity effect through ucp1 expression in white adipose tissues. Biochem Biophys Res Commun.

[71] Chernomorsky S, Segelman A, Poretz RD (1999). Effect of dietary chlorophyll derivatives on mutagenesis and tumor cell growth. Teratog Carcinog Mutagen.

[72] Donaldson MS (2004). Nutrition and cancer: A review of the evidence for an anti-cancer diet. Nutr J.

[73] Li Y, Qian Z-J, Ryu B, Lee S-H, Kim M-M, Kim S-K (2009). Chemical components and its antioxidant properties *in vitro*: An edible marine brown alga *ecklonia cava*. Bioorg Med Chem.

[74] Wang T, Jónsdóttir R, Ólafsdóttir G (2009). Total phenolic compounds, radical scavenging and metal chelation of extracts from icelandic seaweeds. Food Chem.

[75] Bhakuni DS, Rawat DS (2005). Bioactive Marine Natural Products.

[76] Mata TM, Martins AA, Caetano NS (2010). Microalgae for biodiesel production and other applications: A review. Renew Sust Energ Rev.

[77] Guil-Guerrero J, Navarro-Juárez R, López-Martínez J, Campra-Madrid P, Rebolloso-Fuentes M (2004). Functional properties of the biomass of three microalgal species. J Food Eng.

[78] Spolaore P, Joannis-Cassan C, Duran E, Isambert A (2006). Commercial applications of microalgae. J Biosci Bioeng.

[79] Sheih IC, Wu T-K, Fang TJ (2009). Antioxidant properties of a new antioxidative peptide from algae protein waste hydrolysate in different oxidation systems. Bioresour Technol.

[80] Metting FB (1996). Biodiversity and application of microalgae. J Ind Microbiol.

[81] Herrero M, Ibáñez E, Cifuentes A, Reglero G, Santoyo S (2006). *Dunaliella salina* microalga pressurized liquid extracts as potential antimicrobials. J Food Prot.

[82] Mendiola JA, Jaime L, Santoyo S, Reglero G, Cifuentes A, Ibañez E, Señoráns F (2007). Screening of functional compounds in supercritical fluid extracts from spirulina platensis. Food Chem.

[83] Huheihel M, Ishanu V, Tal J, Arad S (2002). Activity of *porphyridium* sp. Polysaccharide against herpes simplex viruses *in vitro* and *in vivo*. J Biochem Biophys Methods.

[84] Kanekiyo K, Lee J-B, Hayashi K, Takenaka H, Hayakawa Y, Endo S, Hayashi T (2005). Isolation of an antiviral polysaccharide, nostoflan, from a terrestrial cyanobacterium *nostoc flagelliforme*. J Nat Prod.

[85] Yeum K-J, Russell RM (2002). Carotenoid bioavailability and bioconversion. Annu Rev Nutr.

[86] Miyashita K (2009). Function of marine carotenoids. Forum Nutr.

[87] Dufossé L, Galaup P, Yaron A, Arad SM, Blanc P, Chidambara Murthy KN, Ravishankar GA (2005). Microorganisms and microalgae as sources of pigments for food use: A scientific oddity or an industrial reality. Trends Food Sci Technol.

[88] Olson JA, Krinsky NI (1995). Introduction: The colourful, fascinating world of the carotenoids: Important physiologic modulators. FASEB J.

[89] Guerin M, Huntley ME, Olaizola M (2003). Haematococcus astaxanthin: Applications for human health and nutrition. Trends Biotechnol.

[90] Undeland I, Lindqvust H, Chen-Yun Y, Falch E, Ramel A, Cooper M, Gildberg A, Luten J, Stenberg E, Nielsen HH, Elvevoll E, Luten J (2009). Seafood and health: What is the full story?. Marine Functional Food.

[91] Kelleher K (2005). Discards in the World’s Marine Fisheries An Update.

[92] Rustad T (2003). Utilisation of marine by-products. EJEAFChe.

[93] Ferraro V, Cruz IB, Jorge RF, Malcata FX, Pintado ME, Castro PML (2010). Valorisation of natural extracts from marine source focused on marine by-products: A review. Food Res Int.

[94] Jeon Y-J, Kim S-K (2002). Antitumor activity of chitosan oligosaccharides produced in ultrafiltration membrane reactor system. J Microbiol Biotechnol.

[95] Je J-Y, Park P-J, Kim S-K (2005). Antioxidant activity of a peptide isolated from alaska pollack (*theragra chalcogramma*) frame protein hydrolysate. Food Res Int.

[96] Kim S-K, Mendis E (2006). Bioactive compounds from marine processing byproducts-a review. Food Res Int.

[97] Fujita H, Yoshikawa M (1999). Lkpnm: A prodrug-type ace-inhibitory peptide derived from fish protein. Immunopharmacology.

[98] Je J-Y, Park P-J, Kwon JY, Kim S-K (2004). A novel angiotensin I converting enzyme inhibitory peptide from alaska pollack (*theragra chalcogramma*) frame protein hydrolysate. J Agric Food Chem.

[99] Rajapakse N, Jung W-K, Mendis E, Moon S-H, Kim S-K (2005). A novel anticoagulant purified from fish protein hydrolysate inhibits factor xiia and platelet aggregation. Life Sci.

[100] Jun S-Y, Park P-J, Jung W-K, Kim S-K (2004). Purification and characterization of an antioxidative peptide from enzymatic hydrolysate of yellowfin sole (*limanda aspera*) frame protein. Eur Food Res Technol.

[101] Jung W-K, Park P-J, Byun H-G, Moon S-H, Kim S-K (2005). Preparation of hoki (johnius belengerii) bone oligophosphopeptide with a high affinity to calcium by carnivorous intestine crude proteinase. Food Chem.

[102] Choi SS, Regenstein JM (2000). Physicochemical and sensory characteristics of fish gelatin. J Food Sci.

[103] Gómez-Guillén MC, Turnay J, Fernández-Diaz MD, Ulmo N, Lizarbe MA, Montero P (2002). Structural and physical properties of gelatin extracted from different marine species: A comparative study. Food Hydrocolloids.

[104] Nicholson J, Wolmarans M, Park G (2000). The role of albumin in critical illness. Br J Anaesth.

[105] Falch E, Rustad T, Aursand M (2006). By-products from gadiform species as raw material for production of marine lipids as ingredients in food or feed. Process Biochem.

[106] Meyers MA, Chen P-Y, Lin AY-M, Seki Y (2008). Biological materials: Structure and mechanical properties. Prog Mater Sci.

[107] Shahidi F, Arachchi JKV, Jeon Y-J (1999). Food applications of chitin and chitosans. Trends Food Sci Technol.

[108] Martínez-Valverde I, Jesús Periago M, Santaella M, Ros G (2000). The content and nutritional significance of minerals on fish flesh in the presence and absence of bone. Food Chem.

[109] Lloret J (2010). Human health benefits supplied by mediterranean marine biodiversity. Mar Pollut Bull.

[110] Leary D, Vierros M, Hamon G, Arico S, Monagle C (2009). Marine genetic resources: A review of scientific and commercial interest. Mar Policy.

[111] Penesyan A, Kjelleberg S, Egan S (2010). Development of novel drugs from marine surface associated microorganisms. Mar Drugs.

[112] Bajpai P, Bajpai PK (1993). Eicosapentaenoic acid (epa) production from microorganisms: A review. J Biotechnol.

[113] Guezennec J (2002). Deep-sea hydrothermal vents: A new source of innovative bacterial exopolysaccharides of biotechnological interest. J Ind Microbiol Biotechnol.

[114] Dharmaraj S, Ashokkumar B, Dhevendaran K (2009). Food-grade pigments from streptomyces sp. Isolated from the marine sponge callyspongia diffusa. Food Res Int.

[115] Luiten EEM, Akkerman I, Koulman A, Kamermans P, Reith H, Barbosa MJ, Sipkema D, Wijffels RH (2003). Realizing the promises of marine biotechnology. Biomol Eng.

[116] Schwager J, Mohajeri MH, Fowler A, Weber P (2008). Challenges in discovering bioactives for the food industry. Curr Opin Biotechnol.

[117] Zhu C-F, Li G-Z, Peng H-B, Zhang F, Chen Y, Li Y (2010). Effect of marine collagen peptides on markers of metabolic nuclear receptors in type 2 diabetic patients with/without hypertension. Biomed Environ Sci.

[118] Rajanbabu V, Chen J-Y (2011). Applications of antimicrobial peptides from fish and perspectives for the future. Peptides.

[119] Manna S, Janarthan M, Ghosh B, Rana B, Rana A, Chatterjee M (2010). Fish oil regulates cell proliferation, protect DNA damages and decrease her-2/neu and c-myc protein expression in rat mammary carcinogenesis. Clin Nutr.

[120] Mandal CC, Ghosh-Choudhury T, Yoneda T, Choudhury GG, Ghosh-Choudhury N (2010). Fish oil prevents breast cancer cell metastasis to bone. Biochem Biophys Res Commun.

[121] Hubbard NE, Lim D, Erickson KL (1998). Alteration of murine mammary tumorigenesis by dietary enrichment with n-3 fatty acids in fish oil. Cancer Lett.

[122] Karmali RA, Adams L, Trout JR (1993). Plant and marine n-3 fatty acids inhibit experimental metastasis of rat mammary adenocarcinoma cells. Prostaglandins Leukot Essent Fatty Acids.

[123] Emelyanov A, Fedoseev G, Krasnoschekova O, Abulimity A, Trendeleva T, Barnes P (2002). Treatment of asthma with lipid extract of new zealand green-lipped mussel: A randomised clinical trial. Eur Respir J.

[124] Judé S, Roger S, Martel E, Besson P, Richard S, Bougnoux P, Champeroux P, Le Guennec J-Y (2006). Dietary long-chain omega-3 fatty acids of marine origin: A comparison of their protective effects on coronary heart disease and breast cancers. Prog Biophys Mol Biol.

[125] He K (2009). Fish, long-chain omega-3 polyunsaturated fatty acids and prevention of cardiovascular disease--eat fish or take fish oil supplement. Prog Cardiovasc Dis.

[126] Bouldrault C, Bazinet RP, Ma DWL (2009). Experimental models and mechanisms underlying the protective effects of n-3 polyunsaturated fatty acids in alzheimer's disease. J Nutr Biochem.

[127] Cunnane SC, Plourde M, Pifferi F, Bégin M, Féart C, Barberger-Gateau P (2009). Fish, docosahexaenoic acid and alzheimer's disease. Prog Lipid Res.

[128] Itoh H, Noda H, Amano H, Zhuaug C, Mizuno T, Ito H (1993). Antitumor activity and immunological properties of marine algal polysaccharides, especially fucoidan, prepared from *sargassum thunbergii* of phaeophyceae. Anticancer Res.

[129] Wijesekara I, Pangestuti R, Kim S-K (2011). Biological activities and potential health benefits of sulfated polysaccharides derived from marine algae. Carbohydr Polym.

[130] Mendis E, Kim M-M, Rajapakse N, Kim S-K (2007). An *in vitro* cellular analysis of the radical scavenging efficacy of chitooligosaccharides. Life Sci.

[131] Godard M, Décordé K, Ventura E, Soteras G, Baccou J-C, Cristol J-P, Rouanet J-M (2009). Polysaccharides from the green alga *ulva rigida* improve the antioxidant status and prevent fatty streak lesions in the high cholesterol fed hamster, an animal model of nutritionally-induced atherosclerosis. Food Chem.

[132] Thomes P, Rajendran M, Pasanban B, Rengasamy R (2010). Cardioprotective activity of *cladosiphon okamuranus* fucoidan against isoproterenol induced myocardial infarction in rats. Phytomedicine.

[133] Huang L, Wen K, Gao X, Liu Y (2010). Hypolipidemic effect of fucoidan from laminaria japonica in hyperlipidemic rats. Pharm Biol.

[134] Cha KH, Koo SY, Lee D-U (2008). Antiproliferative effects of carotenoids extracted from *chlorella ellipsoidea* and *chlorella vulgaris* on human colon cancer cells. J Agric Food Chem.

[135] Sun Z, Peng X, Liu J, Fan K-W, Wang M, Chen F (2010). Inhibitory effects of microalgal extracts on the formation of advanced glycation endproducts (ages). Food Chem.

[136] Nwosu F, Morris J, Lund VA, Stewart D, Ross HA, McDougall GJ (2011). Anti-proliferative and potential anti-diabetic effects of phenolic-rich extracts from edible marine algae. Food Chem.

[137] Celikler S, Tas S, Vatan O, Ziyanok-Ayvalik S, Yildiz G, Bilaloglu R (2009). Anti-hyperglycemic and antigenotoxic potential of *ulva rigida* ethanolic extract in the experimental diabetes mellitus. Food Chem Toxicol.

[138] Kang C, Jin YB, Lee H, Cha M, Sohn E-t, Moon J, Park C, Chun S, Jung E-S, Hong J-S, Kim SB, Kim J-S, Kim E (2010). Brown alga *ecklonia cava* attenuates type 1 diabetes by activating ampk and akt signaling pathways. Food Chem Toxicol.

[139] Newman D, Cragg G (2004). Advanced preclinical and clinical trials of natural products and related compounds from marine sources. Curr Med Chem.

[140] Mayer AMS, Gustafson KR (2008). Marine pharmacology in 2005–2006: Antitumour and cytotoxic compounds. Eur J Cancer.

[141] Carter N, Keam S (2007). Trabectedin: A review of its use in the management of soft tissue sarcoma and ovarian cancer. Drugs.

[142] Villa FA, Gerwick L (2010). Marine natural product drug discovery: Leads for treatment of inflammation, cancer, infections, and neurological disorders. Immunopharmacol Immunotoxicol.

[143] Rinehart K (2000). Antitumor compounds from tunicates. Med Res Rev.

[144] Russo A, Piovano M, Lombardo L, Garbarino J, Cardile V (2008). Lichen metabolites prevent uv light and nitric oxide-mediated plasmid DNA damage and induce apoptosis in human melanoma cells. Life Sci.

[145] Sipkema D, Franssen MCR, Osinga R, Tramper J, Wijffels RH (2005). Marine sponges as pharmacy. Mar Biotechnol.

[146] Sakowicz R, Beredelis M, Ray K, Blackburn C, Hopmann C, Faulkner D, Goldstein L (1998). A marine natural product inhibitor of kinesin motors. Science.

[147] Prado MP, Torres YR, Berlinck RGS, Desiderá C, Sanchez MA, Craveiro MV, Hajdu E, da Rocha RM, Machado-Santelli GM (2004). Effects of marine organisms extracts on microtubule integrity and cell cycle progression in cultured cells. J Exp Mar Biol Ecol.

[148] Coué M, Brenner SL, Spector I, Korn ED (1987). Inhibition of actin polymerization by latrunculin a. FEBS Lett.

[149] Bubb M, Spector I, Bershadsky A, Korn ED (1995). Swinholide a is a microfilament disrupting marine toxin that stabilizes actin dimers and severs actin filaments. J Biol Chem.

[150] Inaba K, Sato H, Tsuda M, Kobayashi J (1998). Spongiacidins a-d, new bromopyrrole alkaloids from *hymeniacidon* sponge. J Nat Prod.

[151] Soni R, Muller L, Furet P, Schoepfer J, Stephan C, Zumstein-Mecker S, Fretz H, Chaudhuri B (2000). Inhibition of cyclin-dependent kinase 4 (cdk4) by fascaplysin, a marine natural product. Biochem Biophys Res Commun.

[152] Burres N, Clement J (1989). Antitumor activity and mechanism of action of the novel marine natural products mycalamide-a and -b and onnamide. Cancer Res.

[153] Fukuoka K, Yamagishi T, Ichihara T, Nakaike S, Iguchi K, Yamada Y, Fukumoto H, Yoneda T, Samata K, Ikeya H, Nanaumi K, Hirayama N, Narita N, Saijo N, Nishio K (2000). Mechanism of action of aragusterol a (yta0040), a potent anti-tumor marine steroid targeting the g(1) phase of the cell cycle. Int J Cancer.

[154] Marshall KM, Matsumoto SS, Holden JA, Concepción GP, Tasdemir D, Ireland CM, Barrows LR (2003). The anti-neoplastic and novel topoisomerase ii-mediated cytotoxicity of neoamphimedine, a marine pyridoacridine. Biochem Pharmacol.

[155] Juagdan EG, Kalidindi RS, Scheuer PJ, Kelly-Borges M (1995). Elenic acid, an inhibitor of topoisomerase ii, from a sponge, plakinastrella sp. Tetrahedron Lett.

[156] Fung FMY, Ding JL (1998). A novel antitumour compound from the mucus of a coral, galaxea fascicularis, inhibits topoisomerase i and ii. Toxicon.

[157] Chen A, Liu L (1994). DNA topoisomerases: Essential enzymes and lethal targets. Annu Rev Pharmacol Toxicol.

[158] Pardo B, Paz-Ares L, Tabernero J, Ciruelos E, García M, Salazar R, López A, Blanco M, Nieto A, Jimeno J, Izquierdo M, Trigo J (2008). Phase i clinical and pharmacokinetic study of kahalalide f administered weekly as a 1-hour infusion to patients with advanced solid tumors. Clin Cancer Res.

[159] Provencio M, Sánchez A, Gasent J, Gómez P, Rosell R (2009). Cancer treatments: Can we find treasures at the bottom of the sea. Clin Lung Cancer.

[160] Haijin M, Xiaolu J, Huashi G (2003). A k-carrageenan derived oligosaccharide prepared by enzymatic degradation containing anti-tumor activity. J Appl Phycol.

[161] Yuan H, Song J, Li X, Li N, Dai J (2006). Immunomodulation and antitumor activity of [kappa]-carrageenan oligosaccharides. Cancer Lett.

[162] Hiroishi S, Sugie K, Yoshida T, Morimoto J, Taniguchi Y, Imai S, Kurebayashi J (2001). Antitumor effects of marginisporum crassissimum (rhodophyceae), a marine red alga. Cancer Lett.

[163] Zhou G, Sun Y, Xin H, Zhang Y, Li Z, Xu Z (2004). *In vivo* antitumor and immunomodulation activities of different molecular weight lambda-carrageenans from chondrus ocellatus. Pharmacol Res.

[164] de Sousa APA, Torres MR, Pessoa C, deMoraes MO, Filho FDR, Alves APNN, Costa-Lotufo LV (2007). *In vivo* growth-inhibition of sarcoma 180 tumor by alginates from brown seaweed sargassum vulgare. Carbohydr Polym.

[165] Bougnoux P (1999). n-3 polyunsaturated fatty acids and cancer. Curr Opin Clin Nutr Metab Care.

[166] Rose DP, Connolly JM (1999). Omega-3 fatty acids as cancer chemopreventive agents. Pharmacol Ther.

[167] Senzaki H, Iwamoto S, Ogura E, Kiyozuka Y, Arita S, Kurebayashi J, Takada H, Hioki K, Tsubura A (1998). Dietary effects of fatty acids on growth and metastasis of kpl-1 human breast cancer cells *in vivo* and *in vitro*. Anticancer Res.

[168] Brown M, Hart C, Gazi E, Bagley S, Clarke N (2006). Promotion of prostatic metastatic migration towards human bone marrow stoma by omega 6 and its inhibition by omega 3 pufas. Br J Cancer.

[169] Raghuveer C, Tandon R (2009). Consumption of functional foods and our health concerns. Pak J Physiol.

[170] Das SK, Hashimoto T, Kanazawa K (2008). Growth inhibition of human hepatic carcinoma hepg2 cells by fucoxanthin is associated with down-regulation of cyclin d. Biochim Biophys Acta.

[171] Gunasekera R, Sewgobind K, Desai S, Dunn L, Black H, McKeehan W, Patil B (2007). Lycopene and lutein inhibit proliferation in rat prostate carcinoma cells. Nutr Cancer.

[172] Liu C-L, Huang Y-S, Hosokawa M, Miyashita K, Hu M-L (2009). Inhibition of proliferation of a hepatoma cell line by fucoxanthin in relation to cell cycle arrest and enhanced gap junctional intercellular communication. Chem Biol Interact.

[173] Narisawa T, Fukaura Y, Hasebe M, Ito M, Aizawa R, Murakoshi M, Uemura S, Khachik F, Nishino H (1996). Inhibitory effects of natural carotenoids, [alpha]-carotene, [beta]-carotene, lycopene and lutein, on colonic aberrant crypt foci formation in rats. Cancer Lett.

[174] Kim J, Araki S, Kim D, Park C, Takasuka N, Baba-Toriyama H, Ota T, Nir Z, Khachik F, Shimidzu N, Tanaka Y, Osawa T, Uraji T, Murakoshi M, Nishino H, Tsuda H (1998). Chemopreventive effects of carotenoids and curcumins on mouse colon carcinogenesis after 1,2-dimethylhydrazine initiation. Carcinogenesis.

[175] van Poppel G (1993). Carotenoids and cancer: An update with emphasis on human intervention studies. Eur J Cancer.

[176] Tapiero H, Townsend DM, Tew KD (2004). The role of carotenoids in the prevention of human pathologies. Biomed Pharmacother.

[177] Tanaka T, Morishita Y, Suzui M, Kojima T, Okumura A, Mori H (1994). Chemoprevention of mouse urinary bladder carcinogenesis by the naturally occurring carotenoid astaxanthin. Carcinogenesis.

[178] Tanaka T, Makita H, Ohnishi M, Mori H, Satoh K, Hara A (1995). Chemoprevention of rat oral carcinogenesis by naturally occurring xanthophylls, astaxanthin and canthaxanthin. Cancer Res.

[179] Tanaka T, Kawamori T, Ohnishi M, Makita H, Mori H, Satoh K, Hara A (1995). Suppression of azoxymethane-induced rat colon carcinogenesis by dietary administration of naturally occurring xanthophylls astaxanthin and canthaxanthin during the postinitiation phase. Carcinogenesis.

[180] Olvera O, Zimmering S, Arceo C, Cruces M (1993). The protective effects of chlorophyllin in treatment with chromium(vi) oxide in somatic cells of drosophila. Mutat Res Lett.

[181] Chung W-Y, Lee J-M, Park M-Y, Yook J-I, Kim J, Chung A-S, Surh Y-J, Park K-K (1999). Inhibitory effects of chlorophyllin on 7,12-dimethylbenz[a]anthracene-induced bacterial mutagenesis and mouse skin carcinogenesis. Cancer Lett.

[182] Lai C-N, Butler MA, Matney TS (1980). Antimutagenic activities of common vegetables and their chlorophyll content. Mutat Res.

[183] Negishi T, Rai H, Hayatsu H (1997). Antigenotoxic activity of natural chlorophylls. Mutat Res.

[184] Negishi T, Arimoto S, Nishizaki C, Hayatsu H (1989). Inhibitory effect of chlorophyll on the genotoxicity of 3-amino-1-methyl-5h-pyrido[4,3-b]indole (trp-p-2). Carcinogenesis.

[185] Balder H, Vogel J, Jansen M, Weijenberg M, van den Brandt P, Westenbrink S, van der Meer R, Goldbohm R (2006). Heme and chlorophyll intake and risk of colorectal cancer in the netherlands cohort study. Cancer Epidemiol Biomarkers Prev.

[186] Ferruzzi MG, Blakeslee J (2007). Digestion, absorption, and cancer preventative activity of dietary chlorophyll derivatives. Nutr Res.

[187] Schoefs B (2002). Chlorophyll and carotenoid analysis in food products. Properties of the pigments and methods of analysis. Trends Food Sci Technol.

[188] Oben J, Enonchong E, Kuate D, Mbanya D, Thomas T, Hildreth D, Ingolia T, Tempesta M (2007). The effects of proalgazyme novel algae infusion on metabolic syndrome and markers of cardiovascular health. Lipids Health Dis.

[189] Erkkilä AT, Herrington DM, Mozaffarian D, Lichtenstein AH (2005). Cereal fiber and whole-grain intake are associated with reduced progression of coronary-artery atherosclerosis in postmenopausal women with coronary artery disease. Am Heart J.

[190] Mozaffarian D, Kumanyika S, Lemaitre R, Olson J, Burke G, Siscovick D (2003). Cereal, fruit, and vegetable fiber intake and the risk of cardiovascular disease in elderly individuals. J Am Med Assoc.

[191] Bazzano L, He J, Ogden L, Loria C, Whelton P (2002). Dietary fiber intake and reduced risk of coronary heart disease in us men and women: The national health and nutrition examination survey i epidemiologic follow-up study. Arch Intern Med.

[192] Liu S, Buring JE, Sesso HD, Rimm EB, Willett WC, Manson JE (2002). A prospective study of dietary fiber intake and risk of cardiovascular disease among women. J Am Coll Cardiol.

[193] Wolk A, Manson J, Stampfer M, Colditz G, Hu F, Speizer F, Hennekens C, Willett W (1999). Long-term intake of dietary fiber and decreased risk of coronary heart disease among women. J Am Med Assoc.

[194] Venugopal V (2009). Marine Products for Healthcare Functional and Bioactive Nutraceutical Compounds from the Ocean.

[195] Cherng J-Y, Shih M-F (2005). Preventing dyslipidemia by chlorella pyrenoidosa in rats and hamsters after chronic high fat diet treatment. Life Sci.

[196] Wong KH, Sam SW, Cheung PCK, Ang PO (1999). Changes in lipid profiles of rats fed with seaweed-based diets. Nutr Res.

[197] Kimura Y, Watanabe K, Okuda H (1996). Effects of soluble sodium alginate on cholesterol excretion and glucose tolerance in rats. J Ethnopharmacol.

[198] Bang H, Dyerberg J (1972). Plasma lipids and lipoproteins in greenlandic west coast eskimos. Acta Med Scand.

[199] Kromhout D, Bosschieter E, de Lezenne Coulander C (1985). The inverse relation between fish consumption and 20-year mortality from coronary heart disease. N Engl J Med.

[200] William H (2008). Omega-3 fatty acids: The “Japanese” Factor. J Am Coll Cardiol.

[201] Lee J, O'Keefe J, Lavie C, Marchioli R, Harris W (2008). Omega-3 fatty acids for cardioprotection. Mayo Clin Proc.

[202] Burr ML, Gilbert JF, Holliday RM, Elwood PC, Fehily AM, Rogers S, Sweetnam PM, Deadman NM (1989). Effects of changes in fat, fish, and fibre intakes on death and myocardial reinfarction: Diet and reinfarction trial (dart). Lancet.

[203] Oomen C, Feskens E, Räsänen L, Fidanza F, Nissinen A, Menotti A, Kok F, Kromhout D (2000). Fish consumption and coronary heart disease mortality in finland, italy, and the netherlands. Am J Epidemiol.

[204] Lavie CJ, Milani RV, Mehra MR, Ventura HO (2009). Omega-3 polyunsaturated fatty acids and cardiovascular diseases. J Am Coll Cardiol.

[205] Lee J, O'Keefe J, Lavie CJ, Harris W (2009). Omega-3 fatty acids: Cardiovascular benefits, sources and sustainability. Nat Rev Cardiol.

[206] Psota TL, Gebauer SK, Kris-Etherton P (2006). Dietary omega-3 fatty acid intake and cardiovascular risk. Am J Cardiol.

[207] Singh R, Niaz M, Sharma J, Kumar R, Rastogi V, Moshiri M (1997). Randomized, double-blind, placebo-controlled trial of fish oil and mustard oil in patients with suspected acute myocardial infarction: The indian experiment of infarct survival--4. Cardiovasc Drugs Ther.

[208] GISSI-Prevenzione Investigators (1999). Dietary supplementation with n-3 polyunsaturated fatty acids and vitamin e after myocardial infarction: Results of the gissi-prevenzione trial. Lancet.

[209] McLennan P, Howe P, Abeywardena M, Muggli R, Raederstorff D, Mano M, Rayner T, Head R (1996). The cardiovascular protective role of docosahexaenoic acid. Eur J Pharmacol.

[210] McLennan PL, Abeywardena MY, Charnock JS (1988). Dietary fish oil prevents ventricular fibrillation following coronary artery occlusion and reperfusion. Am Heart J.

[211] Charnock JS, McLennan PL, Sundram K, Abeywardena MY (1991). Omega-3 pufa's reduce the vulnerability of the rat heart to ischaemic arrhythmia in the presence of a high intake of saturated animal fat. Nutr Res.

[212] Harris WS, von Schacky C (2004). The omega-3 index: A new risk factor for death from coronary heart disease. Prev Med.

[213] Lerman RH, Kaskel L, McIntosh M, Najm W, Fernandez ML, Baruffi E, Harris W (2011). Correction of the omega-3 index in women with metabolic syndrome by adding omega-3 supplements to a mediterranean style diet. J Clin Lipidol.

[214] Knapp HR, Fitzgerald GA (1989). The antihypertensive effects of fish oil. A controlled study of polyunsaturated fatty acid supplements in essential hypertension. N Engl J Med.

[215] Bønaa KH, Bjerve KS, Straume B, Gram IT, Thelle D (1990). Effect of eicosapentaenoic and docosahexaenoic acids on blood pressure in hypertension. A population-based intervention trial from the tromsø study. N Engl J Med.

[216] Toft I, Bønaa KH, Ingebretsen OC, Nordøy A, Jenssen T (1995). Effects of n-3 polyunsaturated fatty acids on glucose homeostasis and blood pressure in essential hypertension. A randomized, controlled trial. Ann Intern Med.

[217] WHO; ISH (2003). World health organization (who)/international society of hypertension (ish) statement on management of hypertension. J Hypertens.

[218] Zhao Y, Li B, Dong S, Liu Z, Zhao X, Wang J, Zeng M (2009). A novel ace inhibitory peptide isolated from acaudina molpadioidea hydrolysate. Peptides.

[219] Wang J, Hu J, Cui J, Bai X, Du Y, Miyaguchi Y, Lin B (2008). Purification and identification of a ace inhibitory peptide from oyster proteins hydrolysate and the antihypertensive effect of hydrolysate in spontaneously hypertensive rats. Food Chem.

[220] Je J-Y, Park P-J, Byun H-G, Jung W-K, Kim S-K (2005). Angiotensin i converting enzyme (ace) inhibitory peptide derived from the sauce of fermented blue mussel, mytilus edulis. Bioresour Technol.

[221] Jung W-K, Mendis E, Je J-Y, Park P-J, Son BW, Kim HC, Choi YK, Kim S-K (2006). Angiotensin i-converting enzyme inhibitory peptide from yellowfin sole (limanda aspera) frame protein and its antihypertensive effect in spontaneously hypertensive rats. Food Chem.

[222] Lee S-H, Qian Z-J, Kim S-K (2010). A novel angiotensin i converting enzyme inhibitory peptide from tuna frame protein hydrolysate and its antihypertensive effect in spontaneously hypertensive rats. Food Chem.

[223] McNulty H, Jacob RF, Mason RP (2008). Biologic activity of carotenoids related to distinct membrane physicochemical interactions. Am J Cardiol.

[224] Yuan JP, Peng J, Yin K, Wang JH (2010). Potential health-promoting effects of astaxanthin: A high-value carotenoid mostly from microalgae. Mol Nutr Food Res.

[225] Li W, Hellsten A, Jacobsson LS, Blomqvist HM, Olsson AG, Yuan X-M (2004). Alpha-tocopherol and astaxanthin decrease macrophage infiltration, apoptosis and vulnerability in atheroma of hyperlipidaemic rabbits. J Mol Cell Cardiol.

[226] Iwamoto T, Hosoda K, Hirano R, Kurata H, Matsumoto A, Miki W, Kamiyama M, Itakura H, Yamamoto S, Kondo K (2000). Inhibition of low-density lipoprotein oxidation by astaxanthin. J Atheroscler Thromb.

[227] Yoshida H, Yanai H, Ito K, Tomono Y, Koikeda T, Tsukahara H, Tada N (2010). Administration of natural astaxanthin increases serum hdl-cholesterol and adiponectin in subjects with mild hyperlipidemia. Atherosclerosis.

[228] Hussein G, Nakamura M, Zhao Q, Iguchi T, Goto H, Sankawa U, Watanabe H (2005). Antihypertensive and neuroprotective effects of astaxanthin in experimental animals. Biol Pharm Bull.

[229] Hussein G, Goto H, Oda S, Sankawa U, Matsumoto K, Watanabe H (2006). Antihypertensive potential and mechanism of action of astaxanthin: Iii. Antioxidant and histopathological effects in spontaneously hypertensive rats. Biol Pharm Bull.

[230] Mayer AMS, Rodríguez AD, Berlinck RGS, Hamann MT (2007). Marine pharmacology in 2003–4: Marine compounds with anthelmintic antibacterial, anticoagulant, antifungal, anti-inflammatory, antimalarial, antiplatelet, antiprotozoal, antituberculosis, and antiviral activities; affecting the cardiovascular, immune and nervous systems, and other miscellaneous mechanisms of action. Comp Biochem Physiol C Toxicol Pharmacol.

[231] Mayer AMS, Rodríguez AD, Berlinck RGS, Hamann MT (2009). Marine pharmacology in 2005–6: Marine compounds with anthelmintic, antibacterial, anticoagulant, antifungal, anti-inflammatory, antimalarial, antiprotozoal, antituberculosis, and antiviral activities; affecting the cardiovascular, immune and nervous systems, and other miscellaneous mechanisms of action. Biochim Biophys Acta.

[232] Schubert R, Kitz R, Beermann C, Rose MA, Baer PC, Zielen S, Boehles H (2007). Influence of low-dose polyunsaturated fatty acids supplementation on the inflammatory response of healthy adults. Nutrition.

[233] Goldberg RJ, Katz J (2007). A meta-analysis of the analgesic effects of omega-3 polyunsaturated fatty acid supplementation for inflammatory joint pain. Pain.

[234] Belluzzi A, Brignola C, Campieri M, Pera A, Boschi S, Miglioli M (1996). Effect of an enteric-coated fish-oil preparation on relapses in crohn's disease. N Engl J Med.

[235] Bennedsen M, Wang X, Willén R, Wadström T, Andersen LP (1999). Treatment of h. Pylori infected mice with antioxidant astaxanthin reduces gastric inflammation, bacterial load and modulates cytokine release by splenocytes. Immunol Lett.

[236] Lee S, Bai S, Lee K, Namkoong S, Na H, Ha K, Han J, Yim S, Chang K, Kwon Y, Lee S, Kim Y (2003). Astaxanthin inhibits nitric oxide production and inflammatory gene expression by suppressing i(kappa)b kinase-dependent nf-kappab activation. Mol Cells.

[237] Macedo RC, Bolin AP, Marin DP, Otton R (2010). Astaxanthin addition improves human neutrophils function: *In vitro* study. Eur J Nutr.

[238] Mahmoud FF, Haines DD, Abul HT, Abal AT, Onadeko BO, Wise JA (2004). *In vitro* effects of astaxanthin combined with ginkgolide b on t lymphocyte activation in peripheral blood mononuclear cells from asthmatic subjects. J Pharmacol Sci.

[239] James MJ, Cleland LG (1997). Dietary n-3 fatty acids and therapy for rheumatoid arthritis. Semin Arthritis Rheum.

[240] Hurst S, Zainal Z, Caterson B, Hughes CE, Harwood JL (2010). Dietary fatty acids and arthritis. Prostaglandins Leukot Essent Fatty Acids.

[241] Stamp LK, James MJ, Cleland LG (2005). Diet and rheumatoid arthritis: A review of the literature. Semin Arthritis Rheum.

[242] Moskowitz RW (2000). Role of collagen hydrolysate in bone and joint disease. Semin Arthritis Rheum.

[243] Hodge L, Salome CM, Peat JK, Haby MM, Xuan W, Woolcock AJ (1996). Consumption of oily fish and childhood asthma risk. Med J Aust.

[244] Oddy WH, de Klerk NH, Kendall GE, Mihrshahi S, Peat JK (2004). Ratio of omega-6 to omega-3 fatty acids and childhood asthma. J Asthma.

[245] Masuev KA (1997). The effect of polyunsaturated fatty acids of the omega-3 class on the late phase of the allergic reaction in bronchial asthma patients. Ter Arkh.

[246] Masuev KA (1997). The effect of polyunsaturated fatty acids on the biochemical indices of bronchial asthma patients. Ter Arkh.

[247] Nagakura T, Matsuda S, Shichijyo K, Sugimoto H, Hata K (2000). Dietary supplementation with fish oil rich in omega-3 polyunsaturated fatty acids in children with bronchial asthma. Eur Respir J.

[248] Broughton KS, Johnson CS, Pace BK, Liebman M, Kleppinger KM (1997). Reduced asthma symptoms with n-3 fatty acid ingestion are related to 5-series leukotriene production. Am J Clin Nutr.

[249] Villani F, Comazzi R, De Maria P, Galimberti M (1998). Effect of dietary supplementation with polyunsaturated fatty acids on bronchial hyperreactivity in subjects with seasonal asthma. Respiration.

[250] Esiri MM (2007). The interplay between inflammation and neurodegeneration in cns disease. J Neuroimmunol.

[251] Layé S (2010). Polyunsaturated fatty acids, neuroinflammation and well being. Prostaglandins Leukot Essent Fatty Acids.

[252] Barberger-Gateau P, Letenneur L, Deschamps V, Pérès K, Dartigues J, Renaud S (2002). Fish, meat, and risk of dementia: Cohort study. BMJ.

[253] Kalmijn S, Launer LJ, Ott A, Witteman JC, Hofman A, Breteler MM (1997). Dietary fat intake and the risk of incident dementia in the rotterdam study. Ann Neurol.

[254] Jin D-Q, Lim CS, Sung J-Y, Choi HG, Ha I, Han J-S (2006). Ulva conglobata, a marine algae, has neuroprotective and anti-inflammatory effects in murine hippocampal and microglial cells. Neurosci Lett.

[255] Jung W-K, Ahn Y-W, Lee S-H, Choi YH, Kim S-K, Yea SS, Choi I, Park S-G, Seo S-K, Lee S-W, Choi I-W (2009). Ecklonia cava ethanolic extracts inhibit lipopolysaccharide-induced cyclooxygenase-2 and inducible nitric oxide synthase expression in bv2 microglia via the map kinase and nf-[kappa]b pathways. Food Chem Toxicol.

[256] Lim CS, Jin D-Q, Sung J-Y, Lee JH, Choi HG, Ha I, Han J-S (2006). Antioxidant and anti-inflammatory activities of the methanolic extract of *neorhodomela aculeate* in hippocampal and microglial cells. Biol Pharm Bull.

[257] Mayer AMS, Rodríguez AD, Berlinck RGS, Fusetani N (2011). Marine pharmacology in 2007–8: Marine compounds with antibacterial, anticoagulant, antifungal, anti-inflammatory, antimalarial, antiprotozoal, antituberculosis, and antiviral activities; affecting the immune and nervous system, and other miscellaneous mechanisms of action. Comp Biochem Physiol C Toxicol Pharmacol.

[258] McCarty MF, Barroso-Aranda J, Contreras F (2010). Oral phycocyanobilin may diminish the pathogenicity of activated brain microglia in neurodegenerative disorders. Med Hypotheses.

[259] Solfrizzi V, D'Introno A, Colacicco AM, Capurso C, Del Parigi A, Capurso S, Gadaleta A, Capurso A, Panza F (2005). Dietary fatty acids intake: Possible role in cognitive decline and dementia. Exp Gerontol.

[260] Solfrizzi V, Frisardi V, Capurso C, D'Introno A, Colacicco AM, Vendemiale G, Capurso A, Panza F (2010). Dietary fatty acids in dementia and predementia syndromes: Epidemiological evidence and possible underlying mechanisms. Ageing Res Rev.

[261] van Gelder BM, Tijhuis M, Kalmijn S, Kromhout D (2007). Fish consumption, n-3 fatty acids, and subsequent 5-y cognitive decline in elderly men: The zutphen elderly study. Am J Clin Nutr.

[262] Dangour AD, Allen E, Elbourne D, Fletcher A, Richards M, Uauy R (2009). Fish consumption and cognitive function among older people in the uk: Baseline data from the opal study. J Nutr Health Aging.

[263] Nurk E, Drevon CA, Refsum H, Solvoll K, Vollset SE, Nygård O, Nygaard HA, Engedal K, Tell GS, Smith AD (2007). Cognitive performance among the elderly and dietary fish intake: The hordaland health study. Am J Clin Nutr.

[264] Kalmijn S, van Boxtel M, Ocké M, Verschuren W, Kromhout D, Launer L (2004). Dietary intake of fatty acids and fish in relation to cognitive performance at middle age. Neurology.

[265] Nakashima Y, Ohsawa I, Konishi F, Hasegawa T, Kumamoto S, Suzuki Y, Ohta S (2009). Preventive effects of chlorella on cognitive decline in age-dependent dementia model mice. Neurosci Lett.

[266] Uauy R, Dangour AD (2006). Nutrition in brain development and aging: Role of essential fatty acids. Nutr Rev.

[267] Dalton A, Wolmarans P, Witthuhn RC, van Stuijvenberg ME, Swanevelder SA, Smuts CM (2009). A randomised control trial in schoolchildren showed improvement in cognitive function after consuming a bread spread, containing fish flour from a marine source. Prostaglandins Leukot Essent Fatty Acids.

[268] Pei X, Yang R, Zhang Z, Gao L, Wang J, Xu Y, Zhao M, Han X, Liu Z, Li Y (2010). Marine collagen peptide isolated from chum salmon (oncorhynchus keta) skin facilitates learning and memory in aged c57bl/6j mice. Food Chem.

[269] Montgomery P, Richardson AJ (2008). Omega-3 fatty acids for bipolar disorder. Cochrane Database Syst Rev.

[270] Freeman MP, Hibbeln JR, Wisner KL, Davis JM, Mischoulon D, Peet M, Keck PEJ, Marangell LB, Richardson AJ, Lake J, Stoll AL (2006). Omega-3 fatty acids: Evidence basis for treatment and future research in psychiatry. J Clin Psychiatry.

[271] Nemets B, Stahl Z, Belmaker RH (2002). Addition of omega-3 fatty acid to maintenance medication treatment for recurrent unipolar depressive disorder. Am J Psychiatry.

[272] Peet M, Horrobin DF (2002). A dose-ranging study of the effects of ethyl-eicosapentaenoate in patients with ongoing depression despite apparently adequate treatment with standard drugs. Arch Gen Psychiatry.

[273] Stoll AL, Severus WE, Freeman MP, Rueter S, Zboyan HA, Diamond E, Cress KK, Marangell LB (1999). Omega 3 fatty acids in bipolar disorder: A preliminary double-blind, placebo-controlled trial. Arch Gen Psychiatry.

[274] Su K-P, Huang S-Y, Chiu C-C, Shen WW (2003). Omega-3 fatty acids in major depressive disorder: A preliminary double-blind, placebo-controlled trial. Eur Neuropsychopharmacol.

[275] Venna VR, Deplanque D, Allet C, Belarbi K, Hamdane M, Bordet R (2009). Pufa induce antidepressant-like effects in parallel to structural and molecular changes in the hippocampus. Psychoneuroendocrinology.

[276] Diers JA, Ivey KD, El-Alfy A, Shaikh J, Wang J, Kochanowska AJ, Stoker JF, Hamann MT, Matsumoto RR (2008). Identification of antidepressant drug leads through the evaluation of marine natural products with neuropsychiatric pharmacophores. Pharmacol Biochem Behav.

[277] Hong S, Wilson MT, Serizawa I, Wu L, Singh N, Naidenko OV, Miura T, Haba T, Scherer DC, Wei J, Kronenberg M, Koezuka Y, Van Kaer L (2001). The natural killer t-cell ligand alpha-galactosylceramide prevents autoimmune diabetes in non-obese diabetic mice. Nat Med.

[278] Sharif S, Arreaza GA, Zucker P, Mi QS, Sondhi J, Naidenko OV, Kronenberg M, Koezuka Y, Delovitch TL, Gombert JM, Leite-De-Moraes M, Gouarin C, Zhu R, Hameg A, Nakayama T, Taniguchi M, Lepault F, Lehuen A, Bach JF, Herbelin A (2001). Activation of natural killer t cells by alpha-galactosylceramide treatment prevents the onset and recurrence of autoimmune type 1 diabetes. Nat Med.

[279] Pascual I, Lopéz A, Gómez H, Chappé M, Saroyán A, González Y, Cisneros M, Charli JL, Chávez M (2007). Screening of inhibitors of porcine dipeptidyl peptidase iv activity in aqueous extracts from marine organisms. Enzyme Microb Technol.

[280] Gokce G, Haznedaroglu MZ (2008). Evaluation of antidiabetic, antioxidant and vasoprotective effects of *posidonia oceanica* extract. J Ethnopharmacol.

[281] Lee YS, Shin KH, Kim BK, Lee S (2004). Anti-diabetic activities of fucosterol from *pelvetia siliquosa*. Arch Pharm Res.

[282] Taouis M, Dagou C, Ster C, Durand G, Pinault M, Delarue J (2002). N-3 polyunsaturated fatty acids prevent the defect of insulin receptor signaling in muscle. Am J Physiol Endocrinol Metab.

[283] Delarue J, Couet C, Cohen R, Bréchot JF, Antoine JM, Lamisse F (1996). Effects of fish oil on metabolic responses to oral fructose and glucose loads in healthy humans. Am J Physiol.

[284] Khanfar MA, Asal BA, Mudit M, Kaddoumi A, El Sayed KA (2009). The marine natural-derived inhibitors of glycogen synthase kinase-3[beta] phenylmethylene hydantoins: *In vitro* and *in vivo* activities and pharmacophore modeling. Bioorg Med Chem.

